# Altered functional connectivity of the amygdaloid input nuclei in adolescents and young adults with autism spectrum disorder: a resting state fMRI study

**DOI:** 10.1186/s13229-015-0060-x

**Published:** 2016-01-28

**Authors:** Annika Rausch, Wei Zhang, Koen V. Haak, Maarten Mennes, Erno J. Hermans, Erik van Oort, Guido van Wingen, Christian F. Beckmann, Jan K. Buitelaar, Wouter B. Groen

**Affiliations:** Department of Cognitive Neuroscience, Radboud University Medical Center Nijmegen, P.O. Box 9101, 6500 HB Nijmegen, The Netherlands; Donders Institute for Brain, Cognition and Behaviour, Centre for Cognitive Neuroimaging, Radboud University, Nijmegen, The Netherlands; MIRA Institute, University of Twente, Enschede, The Netherlands; Department of Psychiatry, Academic Medical Center, University of Amsterdam, Amsterdam, The Netherlands; Centre for Functional MRI of the Brain (FMRIB), University of Oxford, Oxford, United Kingdom; Karakter Child and Adolescent Psychiatry University Centre, Nijmegen, The Netherlands

**Keywords:** Amygdala, Autism spectrum disorder, Centromedial, Connectivity, Input-output, Laterobasal, Nuclei, Social perception, Superficial

## Abstract

**Background:**

Amygdala dysfunction is hypothesized to underlie the social deficits observed in autism spectrum disorders (ASD). However, the neurobiological basis of this hypothesis is underspecified because it is unknown whether ASD relates to abnormalities of the amygdaloid input or output nuclei. Here, we investigated the functional connectivity of the amygdaloid social-perceptual input nuclei and emotion-regulation output nuclei in ASD versus controls.

**Methods:**

We collected resting state functional magnetic resonance imaging (fMRI) data, tailored to provide optimal sensitivity in the amygdala as well as the neocortex, in 20 adolescents and young adults with ASD and 25 matched controls. We performed a regular correlation analysis between the entire amygdala (EA) and the whole brain and used a partial correlation analysis to investigate whole-brain functional connectivity uniquely related to each of the amygdaloid subregions.

**Results:**

Between-group comparison of regular EA correlations showed significantly reduced connectivity in visuospatial and superior parietal areas in ASD compared to controls. Partial correlation analysis revealed that this effect was driven by the left superficial and right laterobasal input subregions, but not the centromedial output nuclei.

**Conclusions:**

These results indicate reduced connectivity of specifically the amygdaloid sensory input channels in ASD, suggesting that abnormal amygdalo-cortical connectivity can be traced down to the socio-perceptual pathways.

**Electronic supplementary material:**

The online version of this article (doi:10.1186/s13229-015-0060-x) contains supplementary material, which is available to authorized users.

## Background

Autism spectrum disorders (ASD) are a group of neurodevelopmental disorders characterized by severe impairments of reciprocal social interaction and verbal and nonverbal communication and by repetitive and stereotyped behaviors [1, 2]. Structural and functional neuroimaging studies have linked a number of brain structures to ASD symptoms [3–6], one of which is the amygdala. The amygdala theory of autism describes this structure as potential key component in the pathogenesis of ASD [7, 8], since it is involved inwfi various aspects of the social brain, such as social cognition, emotion recognition, socio-communicative perception, and the regulation of emotional responses [9]. In line with this, individuals with ASD tend to show abnormal volume enlargements of the amygdala [10, 11] and have overactive amygdalae in response to mildly aversive stimuli [12] and faces [13], while symptom severity in ASD has been found to correlate with amygdala size [10, 14, 15]. Although amygdala impairments likely relate to pathophysiological socio-emotional processes, its subregion-specific amygdalo-cortical abnormalities have not been stratified in ASD. The aim of this study is to advance our understanding of the pathway-specific amygdala involvement and discern sensory input and response output channels separately.

With the last decade’s paradigm shift in neuroimaging from activity assessment within brain structures to connectivity within neural networks, increasing evidence supports the notion of atypical large-scale neural connectivity in ASD. Some authors hypothesized that the brain in ASD is characterized by long distance underconnectivity and local overconnectivity [16]. Indeed, a number of studies reported large-scale underconnectivity with decreased structural, functional, and interhemispheric connectivity [4, 17, 18], and a few functional magnetic resonance imaging (fMRI) studies found local overconnectivity patterns [19–23]. To date, large-scale functional connectivity abnormalities in the autistic brain have been described most conclusively with respect to hyper- or hyposensitivity to perceptual stimuli [24], which may in part occur due to a lack of sensory integration in ASD [4]. Abnormal sensory processing has primarily been found in the auditory [25–27] and visual system [28–30]. Because social functioning requires the selection and integration of many socio-perceptual stimuli simultaneously, perceptual processing abnormalities may in part account for some of the social difficulties in ASD.

Previous fMRI studies on the role of the amygdala in ASD have generally treated it as a single structure, while in fact it is comprised of at least 13 functionally and structurally distinct nuclei, in which three major input and output units can be discerned [31–33]: the centromedial (CM), laterobasal (LB), and superficial (SF) nuclei. Prior work in animals identified the centromedial part as an output area, which regulates cardiovascular control via projections to the brainstem, cerebellum, and hypothalamus [34]. More specifically, the CM subregion generates ascending projections via the forebrain throughout the cortex and descending projections via the hypothalamus to the brainstem [35]. Via these complex pathways, the CM subregion is thought to modulate autonomic, somatic, and endocrine responses to facilitate appropriate behavioral outcome [36]. A recent study in humans mapped projections from the cortical social "aversion network", a network that is situated around the anterior cingulated cortex, onto a functionally defined amygdaloid subdivision that corresponds to the CM subcompartment [37]. This subdivision may therefore be associated with emotion regulation and response preparation in humans as well [38]. The CM subcompartment receives and integrates most of its projections from the LB nuclei, which maintains broad axonal connections to sensory areas. The LB has been linked to multisensory input and emotional learning [9, 38, 39], especially emotional memory [38]. The SF subregion primarily maintains axonal projections to olfactory cortex [38, 40, 41], and it was found to be more sensitive to emotional face recognition than LB and CM [42], as well as to maintain the most behavioral correlates of the three amygdaloid subregions [38]. The LB and SF comprise structurally and functionally clearly differentiable properties: while the LB mainly receives multisensory environmental input, the SF is thought to receive socially relevant information [43]. Yet, they are often mentioned together as the olfactory/multimodal pallial section of the amygdala as both structures generally process incoming stimuli [32], so as to facilitate social-perceptual processing [44].

Our study specifically aimed to investigate global networks in ASD. Since we consider amygdalo-cortical connectivity long-range connectivity, our hypotheses were aimed at underconnectivity. Given the prominent role of the amygdala in the social brain and previous findings of long-range underconnectivity in sensory areas in ASD, we hypothesized to find reduced functional amygdalo-cortical connectivity in ASD, especially among the projections from sensory cortex to the amygdala. We applied dual-echo imaging and a stringent correction for heart rate and respiratory signals to ensure optimal sensitivity in both the amygdala and neocortical structures.

## Methods

### Participants

Twenty-one adolescents with autistic disorder and 25 typically developing controls were enrolled in the study. We only included participants with an intelligence quotient (full-scale IQ) of 80 or higher and excluded those with ASD who had co-morbid psychiatric or neurological conditions including but not limited to attention deficit/hyperactivity disorder (ADHD), depressive disorder, schizophrenia, epilepsy, or history of traumatic brain injury. We ruled out the presence of psychiatric co-morbidity in controls and verified that all participants scored within the normal range using the school-age version of Child Behavior Check List (CBCL/6-18) and Adult Behavior Check List (ABCL/18-59). Controls were matched at the group level on age, sex, and handedness and verbal, performance, and full-scale IQ scores (Table 1). Participants with ASD were recruited through Karakter, Child and Adolescent Psychiatry University Center, Nijmegen. Diagnoses of autistic disorder were based on a series of clinical assessments including a detailed developmental history, clinical observation, medical work-up, and cognitive testing in a multidisciplinary team including a child psychiatrist and clinical psychologist. Diagnoses of autistic disorder were confirmed with the Autism Diagnostic Interview-Revised (ADI-R) [45], assessed by a trained clinician who met research standards. All participants with ASD met DSM-4 criteria for autistic disorder [1]. Participants under the age of 18 completed the Wechsler Intelligence Scale for Children III (WISC-III) [46], while participants above the age of 18 completed the Wechsler Adult Intelligence Scale III (WAIS-III) [47]. All participants also completed the short version of Edinburgh Handedness Inventory [48]. The two groups did not differ in handedness (*p* = 0.17). In addition, all participants and their parents completed the autism spectrum quotient (AQ) about themselves or their child, respectively. The AQ is a validated measure of autism spectrum characteristics found within both the typical population and individuals with a diagnosis of ASD and thus provides a reliable measurement tool for the comparison of autistic traits between our ASD and control sample [49, 50]. None of the participants used medication.

**Table 1:** Subject demographics

	ASD		Control		
Males	*N* = 19 (95 %)		*N* = 22 (88 %)		
Females	*N* = 1 (5 %)		*N* = 3 (12 %)		
	Mean	SD	Mean	SD	*p* value
Total IQ	102.30	13.57	103.72	9.78	0.69
Verbal IQ	101.00	13.37	104.60	11.29	0.35
Performal IQ	105.88	15.81	103.00	15.39	0.56
Age	16.23	3.18	16.11	2.79	0.90
Autism Questionnaire (AQ)					
Participants	21.83	6.13	11.88	3.91	<0.001*
Parents about participant	30.34	7.57	11.74	5.69	<0.001*
Autism Diagnostic Interview (ADI-R)					
ADI-R A (10)	18.25	6.50			
ADI-R B (8)	15.70	5.54			
ADI-R C (3)	4.05	2.31			
ADI-R D (1)	2.65	1.35			

ADI-R thresholds are shown in parentheses. Pearson chi-squared for group by gender was nonsignificant (value = 0.672, *df* = 1, two-sided asymptotic *p* = 0.412)

*p values* indicate results for the independent *t* test statistic. *ADI-R A* social interaction, *B* communication and language, *C* restricted and repetitive behavior, *D* age of onset criterium

*Statistically significant

The study (including the informed consent procedure and all information brochures) was approved by both the regional ethics committee (Commissie Mensgebonden Onderzoek Arnhem Nijmegen) and Karakter’s review board. All participants provided verbal and written informed consent. For underage participants, parents/guardians also provided verbal and written informed consent. The signed consent forms are kept at Karakter, Child and Adolescent Psychiatry Centre Nijmegen, The Netherlands. To ensure an adequate consent procedure, (1) potential participants were provided with simple language brochures (parents/guardians were provided with regular brochures), (2) the study was not advertised with financial or other incentives other than travel reimbursement (after the scanning procedure, all participants did receive 20 euros for participating irrespective of completion), (3) participants were reminded at each phase of the study that they were free to withdraw from participating, (4) only participants with a total IQ of 80 or higher could participate, and (5) all participants practiced once with the scanning procedure in a replicate (dummy) scanner so that they could experience the scanning procedure and make an informed decision on whether or not to participate.

### Image data acquisition

For each participant, we acquired magnetic resonance imaging (MRI) data at the Donders Institute for Brain, Cognition and Behaviour, Centre for Cognitive Neuroimaging in Nijmegen, The Netherlands, using a 3 Tesla Magnetom Trio (Siemens, Erlangen, Germany) with a 32-channel head coil. The entire scanning session lasted approximately 45 min. For each participant, we collected a T1-weighted whole-brain scan (magnetization-prepared rapid acquisition with gradient echo (MPRAGE), inversion time (TI) = 1100 ms, repetition time (TR) = 2300 ms, echo time (TE) = 3.03 ms, flip angle = 8°, field of view (FOV) = 256 × 256 × 192 mm^3^, voxel size = 1 × 1 × 1 mm^3^) and a resting state scan using T2*-weighted dual-echo planar imaging (EPI, TR = 2510 ms, TE1 = 16 ms, TE2 = 36 ms, flip angle = 83°, FOV = 212 × 212 × 119 mm^3^, voxel size = 2 × 2 × 2.5 mm^3^, number of volumes = 400, imaging bandwidth = 1814 Hz/px, grappa acceleration factor = 4). Note that the usage of dual-echo imaging provides optimal sensitivity for blood-oxygen-level-dependent (BOLD) imaging in both subcortical structures such as the amygdala and the neocortex [51]. For the 16-min resting state scan, participants were instructed to lie still within the scanner with their eyes open, while staying awake and focusing on a small white cross presented at the centre of a projection screen. The first five volumes (12.55 s) were discarded to reduce magnetization equilibration effects. Gradient echo field mapping data were also acquired with identical geometry to the EPI data for EPI off-resonance distortion correction (TR = 1020 ms, TE1 = 10 ms, TE2 = 12.46 ms, flip angle = 90°, FOV = 224 × 224 × 191 mm^3^, voxel size = 3.5 × 3.5 × 2 mm^3^). All participants were able to familiarize themselves with scanner setup and scanning procedure through rehearsal in a replicate (dummy) scanner before actual image acquisition. The scanning session further also included a diffusion tensor imaging (DTI) scan (not reported here).

We recorded participants’ heartbeats using the scanner’s built-in photoplethysmograph, placed on the right index finger. Respiration was measured with a pneumatic belt positioned at the level of the abdomen. In order to reduce the potential bias that the heartbeat and respiration have in resting state BOLD correlation studies [52, 53], we used cardiac and respiratory phase regressors, as well as other nuisance regressors in the fMRI time series analysis.

### Preprocessing

All image preprocessing and analyses were performed using FSL (FMRIB Software Library, http://fsl.fmrib.ox.ac.uk/fsl/fslwiki/) [54]. The following pre-statistical processes were applied to the fMRI data: nonbrain removal using Brain Extraction Tool (BET); rigid-body motion correction using MCFLIRT; high-pass temporal filtering (Gaussian-weighted least-squares fitting with frequency cutoff point = 100 s); correction of off-resonance geometric distortions in the EPI data using PRELUDE and FUGUE, using B_0_ field maps derived from the dual-echo gradient echo dataset; artifact removal based on probabilistic ICA (Independent Component Analysis) using MELODIC; spatial normalization to Montreal Neurological Institute (MNI152) 2 mm isotropic atlas space using boundary-based registration (BBR); and FNIRT and Gaussian filtering (full width at half maximum (FWHM) = 6 mm; see the the “Statistical analysis” section). The dual-echo images (TE = 16 and TE = 36) were combined by averaging both echo times. We excluded one participant with ASD due to excessive head movement in terms of frame-wise displacement (max. frame-wise displacement (FD) = 8.7 mm, *M*_fd_ = 0.89 mm), resulting in 20 datasets from the ASD group and 25 datasets from the control group for further analysis (see Additional file 1 for relative frame-wise displacement). To rule out the possibility that differences in movement between the ASD and control group could contribute to the results, we calculated the mean value of frame-wise movement (i.e., the movement of one TR relative to previous TR) for each participant and compared it between the two groups. No group difference was found (*M*_asd_ = 0.10, SD_asd_ = 0.10; *M*_ctrl_ = 0.07, SD_ctrl_ = 0.42; *t*(25) = 1.73, *p* = 0.1).

### Controlling for structured noise

Our preprocessing stream included several steps to limit the influence of structured noise, such as motion artifacts [55], heartbeat [52], and respiration [53]. First, we conducted manual ICA-based artifact removal. The first author visually inspected all the independent component maps for each participant to identify noise components based on the spatial layout of the component maps and the power spectra of the associated time series [56]. We applied nonaggressive denoising with FSL’s fsl_regfilt, i.e., only variance that was uniquely related to the components labeled as noise component (approx. 70 %) was removed.

After ICA-based noise removal and further preprocessing, we conducted nuisance regression modeling the potential effect from motion and physiological noise on the resting state fMRI data. Specifically, we included six rigid body parameters and the eigenvariate of signals over the entire white matter and the cerebrospinal fluid (CSF) in our GLM. Moreover, we calculated 10 cardiac phase regressors, 10 respiratory phase regressors, and 6 other nuisance regressors including heart rate fluctuation (HRF), heart rate variability (HRV), respiration raw data averaged per TR, respiratory amplitude in 9-s window, respiratory frequency in 9-s window, and RVT (frequency times amplitude of respiration, averaged per TR) that are derived from retrospective image correction (RETROICOR) method [57].

### Region of interest selection

**Fig. 1:**
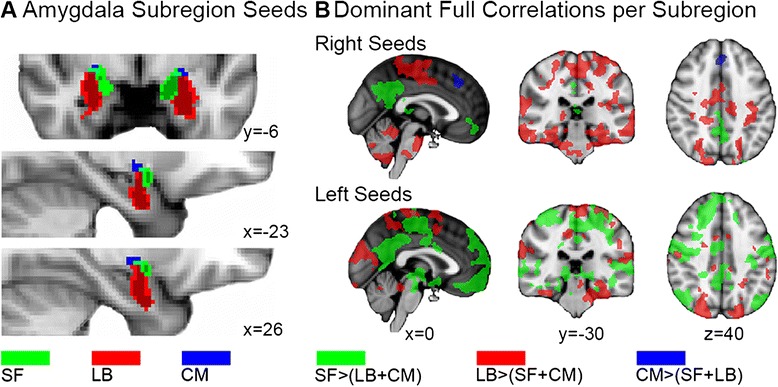
Anatomically defined amygdala regions of interest and its dominant full correlation patterns throughout the cortex. **a** The Juelich cytoarchitectonic histological probability masks of the amygdaloid subregions. *Red areas* depict the laterobasal subregions, *green areas* the superficial subregions, and *blue areas* the centromedial subregions. Areas in *light red*, *light green*, and *dark blue* indicate the 50 % probability mask of each subdivision. Areas in *dark red*, *dark green*, and *light blue* depict the >70 % subregion probability masks that were used for the seed-based analysis. **b** Dominant functional correlations of the left and right amygdala subregions in controls using statistical mean testing. A threshold-free cluster enhancement statistic tested the following contrasts: SF > LB + CM, LB > CM + SF, and CM > LB + SF; (*p* < 0.05, FWE corrected). *Green areas* indicate dominant superficial connectivity networks, *red areas* depict dominant laterobasal networks, and *blue areas* indicate dominant centromedial networks

Stereotaxic, probabilistic maps of the cytoarchitectonic Juelich histological atlas distributed along with FSL were created for the LB (left, 1032 mm^3^; right, 928 mm^3^), centromedial (CM) (left, 16 mm^3^; right, 40 mm^3^), and superficial (SF) (left, 400 mm^3^; right, 160 mm^3^) nuclei (Fig. 1a). The CM part includes the central and medial subdivision. The LB compartment comprises the lateral, basolateral, basomedial, and paralaminar nuclei. The SF subcompartment incorporates the anterior amygdaloid area, the amygdalopyriform transition area, the amygdaloid-hippocampal area, and the ventral and posterior cortical nuclei. Only voxels with a greater than 70 % probability to represent the respective subregion were included in the analysis to reduce overlap between subregions. The entire amygdala region of interest (ROI) was constructed by combining the three amygdaloid subregions into a single structure.

### Statistical analysis

First level analyses were carried out using FSL’s seed-based correlation analysis (SBCA) [58] to calculate the partial correlation between the average time series of the voxels in one ROI (i.e., one of the three amygdala subregions) and the time series of every voxel of the whole brain, corrected for the average time series of the other two amygdala seeds. This tool incorporates the option to add data with different smoothing kernels for the seed and target areas. For the amygdala, cortical correlation analysis we entered spatially unfiltered data for the small amygdaloid areas and FWHM = 6 mm Gaussian filtered functional images for the whole brain. Thus, one single-subject partial correlation map of the brain for each subregion (left and right CM, LB, and SF) was obtained, yielding each subregion’s unique connectivity with the rest of the brain. In addition to the partial correlation analyses, we also performed a regular correlation analysis of the entire amygdala (EA) (left and right) with every voxel in the brain to serve as a reference for the partial correlation results, increasing the interpretability of the partial correlation results.

To test for between-group differences, we performed a nonparametric test with Randomise [59]. As this analysis does not require the data to be normally distributed, an r to z transform is not necessary. Thus, 5000 random permutations of a threshold-free cluster enhancement statistic (TFCE) [60] against the null hypothesis were conducted for each ROI separately. The *p* values were extracted with FSL’s cluster command, where cluster peaks and local maxima with *p* < 0.05 were acquired from the threshold-free cluster enhancement FWE corrected 1-p statistical images of the Randomise output. The permutation method strongly controls for the family-wise error (FWE) rate when a large amount of voxels is tested. Six contrasts (positive main effects in ASD; positive main effects in controls, negative main effects in ASD; negative main effects in controls; ASD > controls; controls > ASD) were tested with an unpaired samples *t* test. The same approach was used for entire amygdala analysis.

The demographic data (IQ, age, and AQ) of both experimental groups were compared using analysis of variance (ANOVA) in SPSS 20 [61] (Table 1).

## Results

### Entire amygdala connectivity

**Fig. 2:**
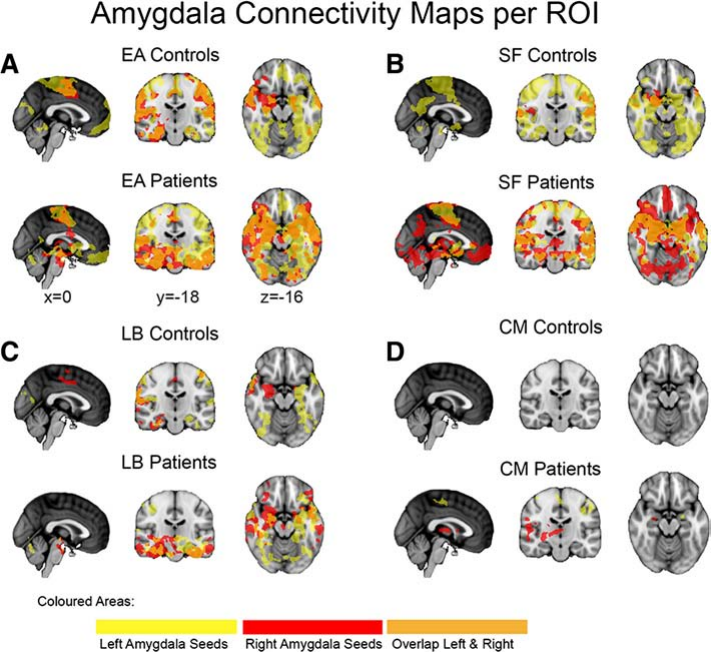
Intrinsic positive connectivity networks of the entire amygdala and its individual subregions in controls and patients. **a** Significant results (*p* < 0.05 FWE corrected) of entire amygdalo-cortical full correlation analyses are delineated for the ASD group (EA patients) and controls (EA controls). *Yellow* and *red areas* depict results from the left and right amygdala seeds, respectively, with *orange regions* illustrating its overlap. Positive main effects (*p* < 0.05 FWE corrected) of the subregion-specific correlation analyses are shown in the same color code for the **b** superficial amygdala in patients (SF patients) and controls (SF controls), **c** the laterobasal amygdala in patients (LB patients) and controls (LB controls) and **d** the centromedial amygdala in patients (CM patients) and controls (CM controls)

To obtain a reference analysis for the subregion-specific amygdala approach and to compare our results with previous literature, we first mapped the intrinsic connectivity of the entire amygdala (Fig. 2a). Only significant clusters surviving family-wise error correction with alpha < 0.05 are reported (Additional file 2).

Figure 2a presents the positive entire amygdala correlations in the ASD and control sample. This reveals a number of symmetric left and right frontal, occipital, temporal, and sensorimotor networks including the cingulate gyrus, which is consistent with the amygdala’s role in socio-emotive circuits [62]. Left amygdala correlations yielded one large cluster with its peak in the left anterior parahippocampal gyrus (−28,−8,−36; *p* < 0.001). Local maxima extended dorsally into the left precentral gyrus, superior parietal lobe, and both sides of the postcentral gyrus and ventrally into the ipsilateral superior temporal gyrus, planum temporale, and temporal pole. Right amygdala correlations were found in the anterior and posterior cingulate gyrus (0,−2,44; *p* = 0.012), including the supplementary motor cortex and clusters in the right temporal fusiform cortex (32,−6,−36; *p* < 0.001), middle temporal gyrus, central opercular cortex, supramarginal gyrus, parietal operculum cortex, a cluster in the left superior temporal gyrus (−62,−8,−4; *p* = 0.002), and a number of smaller clusters in the left inferior temporal gyrus (−46,−58,−16; *p* = 0.036).

The results of the ASD group are comparable with the control group’s results with symmetric left and right frontal, occipital, temporal, and sensorimotor networks underlying emotion regulation circuits (see EA patients in Fig. 2a). The left amygdala revealed a large cluster in the left temporal pole (−26,4,−46; *p* < 0.001) with local maxima in the bilateral pre- and postcentral gyrus. The right amygdala yielded a large cluster in the right temporal pole (28,6,−42; *p* < 0.001) including the local maxima in the temporal areas, the brainstem, and the right hippocampus.

Negative correlations were largely absent in our sample. This is probably a consequence of (1) the participants’ age because during adolescence, negative (inhibitory) mechanisms are not yet as exuberant as after puberty’s transition phase [63–65] and (2) no global signal regression was performed, which could have introduced spurious negative correlations [66, 67] in previous work that did subtract the global mean signal.

### Subregion-specific connectivity

Next, to investigate the individual contributions of the nuclei group-specific seeds, we mapped the intrinsic whole brain connectivity of each ROI (Fig. 2b–d). As before, only significant clusters surviving family-wise error correction with alpha < 0.05 are reported (Additional files 3, 4, 5, and 6).

#### Superficial amygdala connectivity

Left SF connectivity in controls (SF controls, yellow) showed extensive unique bilateral positive correlations throughout frontal (right frontal medial cortex (4,44,−20; *p* = 0.029), left frontal pole (−2,62,−6; *p* = 0.031), temporal (left parahippocampal gyrus (−14,−6,−26; *p* < 0.001), occipital, and parietal lobe and limbic areas including anterior and posterior cingulate gyrus, parahippocampal gyrus, hippocampus, amygdala, thalamus, putamen as well as in the brainstem and cerebellum (Additional files 3 and 6). The right SF showed connectivity to smaller, more selective sensory and limbic areas. In ASD (Fig. 2b, SF patients), SF connectivity yielded a similar pattern, except for a larger cluster in the frontal lobe area, with sparse overall occipital lobe connectivity.

Note that while partial correlation analysis reported for the SF yielded a relatively small frontal lobe cluster in our healthy subjects, a supplementary full correlation analysis revealed a large frontal lobe involvement, indicating that the overlapping signal between SF and CM/LB time series leads to partialling out some of the SF-frontal connectivity (Fig. 1b). Thus, the results indicate that (1) SF functional connectivity in our adolescent sample is consistent with known limbic-striatal-frontal reward related and auditory-parietal-visual valence evaluating circuits [68] and (2) the SF subregion maintains unique whole brain connectivity.

#### Laterobasal amygdala connectivity

The laterobasal subregion exhibited mainly unique connectivity with temporal regions and regions along the lateral superior cortical axis. Although left and right LB connectivity maps overlapped in the parietal operculum cortex, frontal orbital cortex, and temporal pole, strong lateralization effects were also observed (Fig. 2c, LB controls). Left LB connectivity peaks were bilaterally present in the parahippocampal gyrus (left (−26,−8,−34; *p* < 0.001); right (32,−28,−30; *p* = 0.047)), precentral gyrus (left (−44,−14,56; *p* = 0.014)); right (42,−14,52; *p* = 0.017)), and lateral occipital cortex (left (−20,−80,42; *p* = 0.020); right (30,−84,28; *p* = 0.021)) (Additional file 4). Except from one large cluster of connectivity in the right temporal fusiform cortex (32,−6,−36; *p* < 0.001), right LB showed many localized clusters of connectivity, such as in the right supplementary motor area (2,−8,62; *p* = 0.033), right frontal orbital cortex (44,28,−18; *p* = 0.048) and bilateral anterior cingulate gyrus (left (6,−12,44; *p* = 0.031); right (0,−2,44; *p* = 0.033)). In the ASD group, similar patterns of connectivity were observed (Fig. 2c, LB patients). Thus, the LB connectivity maps are in line with its putative involvement in emotional learning through its connection with the (para)hippocampal area and its involvement in multisensory processing via its projections to the sensory systems along the superior temporal gyrus, sensorimotor areas, and visual areas, in combination with cingulate-frontal connectivity [69].

#### Centromedial amygdala connectivity

In the control group, only a small cluster in the right occipital fusiform gyrus (28,−64,−10; *p* = 0.040) from the right centromedial subregion showed a negative correlation, while positive unique functional connectivity did not reach the significance threshold for the left or right CM in the control group using partial correlation analysis (Fig. 2c, CM controls). We therefore performed an additional, regular correlation analysis for the CM to test whether some of its unique signal was “partialled out” due to a functional overlap between CM and LB/SF nuclei (Fig. 1b). As expected, and in line with previous reports [37], positive correlations in anterior cingulate cortex were significantly higher for the CM nucleus compared to SF and LB in healthy controls. This suggests that the partial correlation approach had corrected for the strong overlap with time series from LB and SF to such an extent that the CM’s unique contribution did not reach the FWE-corrected threshold (Additional file 7). The relative lack of unique CM correlations in controls may well reflect the lower degree of functional specialization of the amygdaloid nuclei in adolescents [70]. In the ASD group, however, left CM demonstrated unique partial correlations with bilateral primary sensorimotor areas (postcentral gyrus (left, −36,−22,40; *p* = 0.004), precentral gyrus (right, 40,−14,60; *p* = 0.007)), and left insular cortex in the partial correlation analysis, while right CM correlations with the striatum (including bilateral thalamus, left putamen (−24,0,−10; *p* = 0.046) and right pallidum) and right hemispheric speech processing areas (including central and frontal opercular cortex, supramarginal gyrus, and Heschl’s gyrus) were present (Fig. 2c, CM patients; Additional file 5).

### Difference between ASD and controls

#### Entire amygdala connectivity

**Fig. 3:**
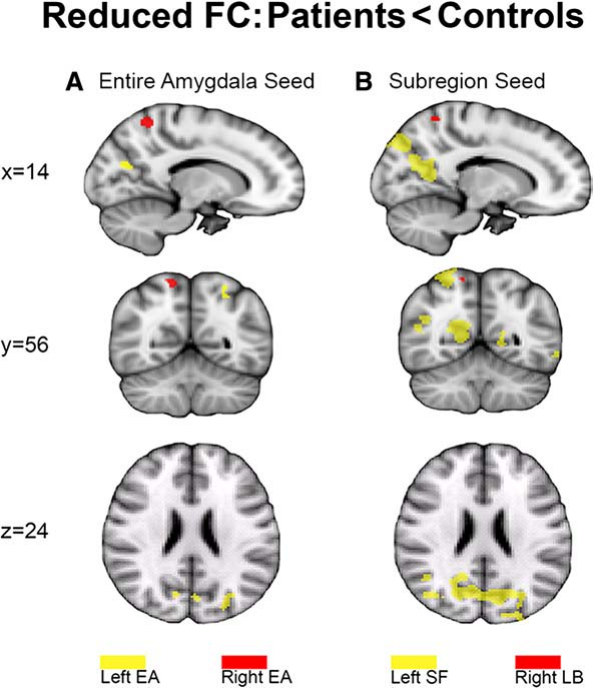
Areas of reduced functional connectivity in ASD. **a** Significant (*p* < 0.05; FWE corrected) reduced connectivity with the entire amygdala (EA) ROI. *Yellow areas* show between-group differences in connectivity with left amygdala seeds, while *red areas* show connectivity with right amygdala seeds. **b** Conventions are depicted as in panel (**a**) but with *yellow regions* illustrating the left superficial ROI and *red* indicating the right laterobasal subcompartment. The results from the partial correlation analysis revealed that the between-group difference in EA was driven by the left SF and right LB. Bilateral CM, right SF, and left LB did not yield significant between-group differences

In line with our hypothesis, we found significantly smaller left amygdala correlations with the left hemispheric occipital pole (−24,−90,34; *p* = 0.026), supracalcarine cortex and intracalcarine cortex, left (−26,−60,58; *p* = 0.040) and right lateral occipital cortex (28,−58,64; *p* = 0.049) in ASD compared to controls. Furthermore, correlations between left amygdala and cuneal cortex were reduced in both hemispheres in ASD. The right amygdala showed significantly reduced correlations with the right superior parietal lobe (14,−54,62; *p* = 0.023) in ASD (Fig. 3a; Additional file 8).

#### Subregion-specific connectivity

To test our hypothesis of reduced amygdala connectivity along sensory input channels in ASD, we directly compared the SF, LB, and CM connectivity between groups (Fig. 3b; Additional file 8). In line with our hypothesis, the ASD group showed reduced left SF connectivity with bilateral precuneus cortex (left, 16,−58,8; *p* = 0.003), cuneal cortex, intracalcarine cortex, lateral occipital cortex (right, 44,−60,12; *p* = 0.023), and supracalcarine cortex in the right hemisphere, the occipital pole, the superior parietal lobe (28,−54,62; *p* = 0.004), and the pre- and postcentral and angular gyri (44,−16,64; *p* = 0.047) when compared with controls. Furthermore, functional connectivity between the right LB and right superior parietal lobe (14, −54, 62; *p* = 0.023) were reduced in ASD. There were no significant between-group differences in the CM correlation maps. Statistical tests in both directions (ASD > controls; controls > ASD) were included in our analysis, but yielded nonsignificant results for ASD overconnectivity patterns. When the analysis was repeated including age as covariate, the group effects were very similar to the previous between-group results and no age-by-group interactions were found. Age showed a negative main effect with connectivity strength in the left SF, indicating that connectivity strength decreases during development in the temporal pole and lateral occipital cortex (Additional file 9).

##### Relation between reduced connectivity strength and AQ scores

To investigate whether the subregion-specific group differences in functional connectivity can be related to specific behavioral/symptomatic characteristics, we quantified the relationship between the participants’ scores within each group on each AQ subdomain and the (z-transformed) correlation between the mean fMRI time series from the right LB and left SF nuclei and the cortical regions that exhibited a significant group difference in functional connectivity. For either group, however, none of the specific AQ sub-domains were significantly related to the subregion-specific functional connectivity strengths. This absence of significance was also observed for the relationship between total AQ score and subregion-specific connectivity strength.

### Subregion signal-to-noise ratios

In theory, the differences in size in the amygdaloid nuclei seed regions might have induced different tSNR (time series’ signal-to-noise ratios) levels between seed regions. To rule out this possibility, we tested whether tSNR varied significantly across seed regions using subject-wise tSNRs from the preprocessed functional images before ICA denoising (Additional file 10). A three-way ANOVA (factors: subregion, diagnostic group, and hemisphere) demonstrated that tSNRs differed between hemispheres (*F* = 8.724, *df* = 1, *p* = 0.003) and diagnostic groups (*F* = 3.770, *df* = 1, *p* = 0.53), but not between subregions (*F* = 0.348, *df* = 2, *p* = 0.707). Furthermore, there were no significant interaction effects between subregions, diagnostic groups, and hemispheres, indicating that differences in subregion tSNR were not affected by diagnostic status or lateralization effects (Table 2). Therefore, the absence of CM main effects in the control group as well as its negative findings in the between-group analysis is not likely caused by different tSNR levels of the CM subregion.

**Table 2:** Analysis of subregion signal-to-noise ratios

Factor	*df*	Mean square	*F*	*p* value
Hemisphere	1	301.406	8.724	0.003*
Subregion	2	12.014	0.348	0.707
Diagnostic group	1	130.24	3.770	0.053
Hemipshere*Subregion	2	18.722	0.542	0.582
Subregion*Diagnostic group	2	1.031	0.030	0.971
Hemisphere*Diagnostic group*Subregion	3	10.098	0.292	0.831

*p values* indicate results for between-subjects effects of tSNR (time-series’ signal-to-noise ratios), *df* degrees of freedom, *F* univariate ANOVA

*statistically significant

## Discussion

In the present study, we investigated amygdalo-cortical connectivity in adolescents and young adults with ASD and controls during resting state fMRI. We found reduced cortical connectivity of both amygdaloid input subregions (SF and LB) with the prefrontal, parietal, and occipital cortices in participants with ASD, while CM output connectivity was spared.

The positive correlation maps from both the entire amygdala and the three subregions in our control group of healthy adolescents revealed large overall overlap with the spontaneous activation maps previously reported in healthy adults [33]. The present results indicate that most of the entire amygdala connectivity main effects can be disentangled into subunit functionality in adolescents and young adults with and without ASD, while some of the global effects (e.g., frontal lobe activation) could not be traced down to one particular subunit. As positive partial CM correlations did not reach FWE-corrected significance in the control group, one interpretation of these findings could entail a lower degree of functional specialization in healthy adolescents. While this may reflect the not yet fully differentiated amygdala in adolescence, the conservative partial correlation approach may also have contributed to the negative finding because normal CM anterior cingulated gyrus connections were found with the full correlation approach. The absence of significant partial correlations for the CM compartment in the controls is most likely due to commonalities (i.e., shared variance) between the CM signals and those from the other subregions, leading them to be partialled out. Indeed, a direct comparison revealed no significant differences between patients and controls. Positive laterobasal-cortical correlations in the prefrontal, parietal, and temporal cortex further confirmed previous findings that connect the LB with associative learning processes across sensory modalities [33, 71], while SF-cortical correlations were in line with known limbic lobe connectivity [33].

Importantly, we found reduced EA connectivity in the ASD group when compared to controls. In line with our hypothesis of amygdala underconnectivity in ASD, we found reduced left EA connectivity with portions of the occipital pole, cuneal cortex, intra- and supracalcarine cortex and lateral occipital cortex in the ASD group. Partial correlation analysis revealed that these differences were largely driven by the left SF subregion, with reduced left SF connectivity in the precuneus, cuneus, angular gyrus, precentral, and postcentral gyrus and superior parietal cortex. The ASD group also exhibited reduced right entire amygdala connectivity with the right superior parietal lobe. Partial correlation analysis revealed that this difference was mainly driven by the right LB.

Prior research showed that functional specialization of amygdaloid subregions continues throughout adolescence [70, 72] and that volumetric abnormalities in ASD are age specific [10, 11]. The possibility that our between-group effects were driven by developmental differences was however not supported by our data. There were no group-by-age interactions for EA and subregion-specific connectivity, and our reported between-group effects were similar to the between-group effects with age as covariate. The negative main effects of age showed no overlap with the abnormalities found for the left SF in our ASD group. Thus, while the age effect in the left superficial area might reflect the developmental changes of the amygdaloid subcompartments throughout adolescence as reported by Gabard-Durnam and colleagues [72] and Qin et al. [70], the amygdala-cortical abnormalities in ASD are not significantly age dependent, at least within the age range examined in the present study.

Although our amygdalo-cortical analysis was not specifically designed for investigating local overconnectivity, increased activation patterns in ASD were also tested and yielded nonsignificant results. Investigating the local overconnectivity account remains challenging in fMRI research [73], and only few studies particularly investigated local overconnectivity in fMRI resting state [19, 21–23] and generated inconsistent results.

Previous task-based fMRI studies have reported some support for pathway-specific deficits of the parietal visuospatial domain in ASD [28, 29, 74] and its connections to the amygdala [75]. Since reduced functional amygdalo-cortical connectivity in our ASD sample was mainly present in the dorso-dorsal and ventro-dorsal pathway, our results suggest that abnormal amygdaloid connectivity in ASD is pathway specific. That is, the fact that we only found abnormal connectivity with the left SF and the right LB, i.e., the amygdaloid input areas, but not with the CM output areas, supports the notion that social-emotional deficits in ASD may be reflected in reduced connectivity along amygdalo-sensory input pathways. Thus, a deficiency specific to the amygdalo-cortical input pathway may account for the social perceptual deficits in ASD.

Our results also showed lateralized subregion-specific amygdaloid connectivity, which contradicts a previous finding of bilateral homogenous amygdala connectivity [33]. However, a number of studies associated the left amygdala with slower explicit emotion appraisal processes, while the right amygdala is more involved with faster implicit threat detection [76]. Another hemispheric lateralization account distinguishes the left hemispheric abstract category subsystem and the right hemispheric whole-based subsystem: the left subsystem uses a parts-based processing strategy to represent smaller features of larger whole objects, while the right hemispheric whole-based subsystem serves visual discrimination of similar objects (see [77]). As the amygdalo-cortical deficits in our ASD sample accumulated along the left SF and right LB sensory input pathways, while other amygdalo-cortical pathways were spared, the results might indicate that the cortical processing of visual object features may be affected in ASD. That is, deficits in the left SF may account for abnormal parts-based perceptual processes along the ventro-dorsal and dorso-dorsal perceptual pathway, while abnormal functional connectivity in the right LB might for instance reflect whole-object face processing difficulties in ASD caused by abnormal amygdalo-cortical connectivity with the right superior parietal lobe.

Within groups, we did not observe a significant relationship between the subregion-specific reductions in functional connectivity strength and the participants’ AQ scores. Given the observed differences in subregion-specific functional connectivity strength across groups, and the fact that ASD status and AQ score are clearly related (see Table 1), it is likely that the absence of statistical significance for these within-group comparisons is related to insufficient statistical power. Future work based on larger sample sizes may therefore be able to tease apart the effects of subregion-specific reductions in functional connectivity on behavior.

Our investigative approach draws on Roy and colleagues’ paper that describes resting state analysis of probabilistic cytoarchitectonically defined amygdala nuclei [33]. In our study, however, partial correlation analysis was used instead of regression analysis with statistical mean testing to compute the unique contributions of each of the three amygdaloid subdivisions, and we studied adolescent brains rather than healthy adult brains. As such, small differences between the two studies may be expected. For instance, in our study, the CM subcompartment showed connectivity with the striatal circuitry in the ASD group, while positive partial correlations did not reach FWE-corrected significance in the control group. Because we did find normal positive correlations in a full correlation analysis for the CM (Fig. 1b), we interpret the lack of partial CM-whole-brain correlations in controls as a reflection of the lower degree of functional specialization of the amygdaloid nuclei in the adolescent brain [70]. Further support for the notion of functional overlap between our three subcompartments was found during supplementary analysis of colinearity (Additional file 7). A putative faster maturation of the CM region in ASD as compared to controls was not supported by the data; we directly tested for increased connectivity patterns in ASD compared to controls but found no difference. Overall, we regard our method as a valid approach for detecting true functional connectivity differences between healthy and clinical populations [78], since (1) the entire amygdala results showed strong overlap with the subregion-specific outcomes from the partial correlation analysis (Additional file 11), (2) the current results clearly demonstrate the expected functional connectivity with known amygdala circuits, and (3) sensitivity to between-group effects increases with partial correlation analysis (Fig. 3, Additional file 8).

## Conclusions

To conclude, the current findings provide further evidence for underconnectivity in socio-emotive circuits in adolescents and young adults with ASD. As we found abnormal connectivity in the amygdala’s input areas but not in the output areas, the findings support the notion that deficient/impaired amygdaloid sensory input mechanisms may underlie ASD. This might indicate that therapeutic interventions should target sensory input channels.

## Additional files

Additional file 1:
**Relative frame-wise displacement.** Indicates movement between fMRI scans in participants with autism spectrum disorder and control subjects. (DOC 41 kb) Additional file 2:
**Intrinsic entire amygdalo-cortical functional connectivity.** Demonstrates main effects of left entire and right entire amygdalo-cortical correlation analysis in participants with autism spectrum disorder and control subjects. (DOC 228 kb) Additional file 3:
**Intrinsic superficial-cortical functional connectivity.** Demonstrates main effects of left and right superficial-cortical partial correlation analysis in participants with Autism Spectrum Disorder and control subjects. (DOC 196 kb) Additional file 4:
**Intrinsic laterobasal-cortical functional connectivity.** Demonstrates main effects of left and right laterobasal-cortical partial correlation analysis in participants with autism spectrum disorder and control subjects. (DOCX 384 kb) Additional file 5:
**Intrinsic centromedial-cortical functional connectivity.** Demonstrates main effects of left and right centromedial-cortical partial correlation analysis in participants with autism spectrum disorder and control subjects. (DOCX 109 kb) Additional file 6:
**Intrinsic amygdalo-cortical functional connectivity in superficial subregion.** Demonstrates a selection of superficial-cortical partial correlation main effects with adjusted significant thresholds in participants with autism spectrum disorder and control subjects. (DOC 253 kb) Additional file 7:
**Functional overlap between the superficial, laterobasal, and centromedial subregions in control subjects.** (DOC 30 kb) Additional file 8:
**Reduced functional connectivity in ASD.** Demonstrates between-group differences of entire amygdalo-cortical correlations and subregion-specific partial correlations between participants with autism spectrum disorder and control subjects. (DOC 107 kb) Additional file 9:
**Functional connectivity with age effects.** Demonstrates between-group differences of entire amygdalo-cortical correlations and nuclei group-specific partial correlations between participants with autism spectrum disorder and control subjects and negative main effects of age in the superficial subcompartment. (DOC 85 kb) Additional file 10:
**Subject-wise tSNR levels per nucleus group.** (DOC 4303 kb) Additional file 11:
**Similarity between all partial correlation maps combined and entire amygdala correlation maps in healthy subjects.** (DOC 55 kb)

## Abbreviations

ABCL/18-59: Adult Behavior Check List/age 18-59
ADI-R: Autism Diagnostic Interview-Revised
ADHD: attention deficit/hyperactivity disorder
ANOVA: analysis of variance
ASD: autism spectrum disorder
AQ: autism spectrum quotient
BBR: boundary-based registration
BET: Brain Extraction Tool
BOLD: blood-oxygen-level dependent
B0: symbol for the constant magnetic field
CBCL/6-18: Child Behavior Check List/age 6-18
CM: centromedial amygdaloid subcompartment
CSF: cerebrospinal fluid
DSM-4: Diagnostic and Statistical Manual of Mental Disorders (4th edition)
DTI: diffusion tensor imaging
EA: entire amygdala
EPI: echo-planar imaging
FC: Functional Connectivity
FD: frame-wise displacement
fMRI: functional magnetic resonance imaging
FMRIB: (Oxford centre for) functional magnetic resonance imaging of the brain
FNIRT: FMRIB’s nonlinear image registration tool
FOV: field of view, spatial encoding area of the image
FSL: FMRIB Software Library
FUGUE: FMRIB's Utility for Geometrically Unwarping EPIs
FWHM: full width at half maximum
HRF: heart rate fluctuation
HRV: heart rate variability
ICA: independent component analysis
IQ: intelligence quotient
LB: laterobasal amygdaloid subcompartment
MCFLIRT: FMRIB's Linear registration and motion correction tool
MELODIC: Multivariate Exploratory Linear Optimized Decomposition into Independent Components
MPRAGE: magnetization-prepared rapid acquisition with gradient echo
MRI: magnetic resonance imaging
PRELUDE: Phase Region Expanding Labeller for Unwrapping Discrete Estimates
RETROICOR: retrospective image correction
ROI: region of interest
RVT: respiration volume per time
SBCA: seed-based correlation analysis
SF: superficial amygdaloid subcompartment
SPSS 20: Statistical Package for the Social Sciences (20th edition)
TE: echo time
TI: inversion time
TR: repetition time
T1-weighted: an image whose contrast and brightness are predominately determined by T1 signals (T1: time constant at which spins realign themselves with the external magnetic field after excitation)
T2*: T-two-star, time constant for the loss of phase coherence among spins oriented at an angle to the static magnetic field
WAIS-III: Wechsler Adult Intelligence Scale (3rd edition)
WISC-III: Wechsler Intelligence Scale for Children (3rd edition)
