# Supplementary material for: Altered functional connectivity of the amygdaloid input nuclei in adolescents and young adults with autism spectrum disorder: a resting state fMRI study
Source: Mol Autism. 2016 Jan 28;7:13. doi: 10.1186/s13229-015-0060-x (PMC4730628; doi:10.1186/s13229-015-0060-x)
Supplement: Additional file 1: — Relative frame-wise displacement. Indicates movement between fMRI scans in participants with autism spectrum disorder and control subjects. (DOC 41 kb) [file 13229_2015_60_MOESM1_ESM.doc]

**Additional file 1. Relative frame-wise displacement.**

| **ASD** | | **Control** | |
| --- | --- | --- | --- |
| **Participant** | **FD** (mm) | **Participant** | **FD** (mm) |
| 1 | 0.35 | 1 | 0.06 |
| 2 | 0.03 | 2 | 0.03 |
| 3 | 0.21 | 3 | 0.04 |
| 4 | 0.11 | 4 | 0.08 |
| 5 | 0.13 | 5 | 0.06 |
| 6 | 0.06 | 6 | 0.08 |
| 7 | 0.11 | 7 | 0.05 |
| 8 | 0.06 | 8 | 0.05 |
| 9 | 0.06 | 9 | 0.06 |
| 10 | 0.37 | 10 | 0.06 |
| 11 | 0.05 | 11 | 0.06 |
| 12 | 0.04 | 12 | 0.07 |
| 13 | 0.06 | 13 | 0.05 |
| 14 | 0.06 | 14 | 0.03 |
| 15 | 0.06 | 15 | 0.07 |
| 16 | 0.06 | 16 | 0.08 |
| 17 | 0.06 | 17 | 0.04 |
| 18 | 0.05 | 18 | 0.11 |
| 19 | 0.10 | 19 | 0.06 |
| 20 | 0.06 | 20 | 0.25 |
|  |  | 21 | 0.03 |
|  |  | 22 | 0.06 |
|  |  | 23 | 0.04 |
|  |  | 24 | 0.06 |
|  |  | 25 | 0.06 |

Additional file 1 shows the frame-wise displacement (FD), i.e. a measure of movement between fMRI scans. There was no significant group difference and none of the participants showed exceeding in-plane voxel-size (2mm) movement.
