# Supplementary material for: Altered functional connectivity of the amygdaloid input nuclei in adolescents and young adults with autism spectrum disorder: a resting state fMRI study
Source: Mol Autism. 2016 Jan 28;7:13. doi: 10.1186/s13229-015-0060-x (PMC4730628; doi:10.1186/s13229-015-0060-x)
Supplement: Additional file 2: — Intrinsic entire amygdalo-cortical functional connectivity. Demonstrates main effects of left entire and right entire amygdalo-cortical correlation analysis in participants with autism spectrum disorder and control subjects. (DOC 228 kb) [file 13229_2015_60_MOESM2_ESM.doc]

**Additional file 2**. Intrinsic entire amygdalo-cortical functional connectivity.

| ***EA Left Controls*** | | | | | |
| --- | --- | --- | --- | --- | --- |
| *positive* | | | | | |
| Cluster Size | Structure | x | y | z | p-value |
| 43211 | Parahippocampal Gyrus (L) | -28 | -8 | -36 | 0.000 |
|  |  | -22 | -8 | -34 | 0.001 |
|  | Postcentral Gyrus (L) | -30 | -36 | 70 | 0.001 |
|  |  | -28 | -40 | 68 | 0.001 |
|  |  | -32 | -32 | 66 | 0.001 |
|  |  | -38 | -26 | 58 | 0.001 |
|  |  | -42 | -18 | 48 | 0.001 |
|  | Postcentral Gyrus (R) | 42 | -32 | 66 | 0.001 |
|  |  | 40 | -26 | 64 | 0.001 |
|  |  | 38 | -32 | 64 | 0.001 |
|  | Superior Parietal Lobule (L) | -38 | -46 | 60 | 0.001 |
|  |  | -32 | -40 | 52 | 0.001 |
|  | Precentral Gyrus (L) | -42 | -20 | 58 | 0.001 |
|  |  | -24 | -28 | 58 | 0.001 |
|  | Planum Temporale (L) | -64 | -28 | 12 | 0.001 |
|  |  | -54 | -22 | 6 | 0.001 |
|  |  | -60 | -16 | 4 | 0.001 |
|  | Superior Temporal Gyrus (L) | -62 | -8 | 2 | 0.001 |
|  | Temporal Pole (L) | -58 | 4 | -16 | 0.001 |
|  |  | -54 | 4 | -30 | 0.001 |
|  |  | -44 | 4 | -30 | 0.001 |
| *negative* | | | | | |
| 35 | Supramarginal Gyrus (R) | 56 | -44 | 50 | 0.031 |
|  |  |  |  |  |  |
| ***EA Right Controls*** | | | | | |
| *positive* | | | | | |
| Cluster Size | Structure | x | y | z | p-value |
| 12392 | Temporal Fusiform Cortex (R) | 32 | -6 | -36 | 0.000 |
|  |  | 26 | -6 | -44 | 0.003 |
|  | Planum Temporale (R) | 62 | -8 | 4 | 0.002 |
|  |  | 62 | -22 | 8 | 0.003 |
|  |  | 62 | -16 | 6 | 0.003 |
|  | Postcentral Gyrus (R) | 54 | -8 | 34 | 0.003 |
|  |  | 62 | -16 | 30 | 0.004 |
|  |  | 58 | -16 | 26 | 0.004 |
|  | Precentral Gyrus (R) | 62 | -4 | 32 | 0.003 |
|  |  | 62 | -2 | 24 | 0.003 |
|  |  | 64 | 4 | 16 | 0.003 |
|  | Central Opercular Cortex (R) | 50 | -16 | 12 | 0.003 |
|  | Superior Temporal Gyrus (R) | 68 | -22 | 10 | 0.003 |
|  | Planum Polare (R) | 60 | 2 | 2 | 0.003 |
|  | Temporal Pole (R) | 60 | 8 | -4 | 0.003 |
|  |  | 42 | 14 | -38 | 0.003 |
|  |  | 30 | 4 | -42 | 0.003 |
|  |  | 52 | 8 | -20 | 0.003 |
|  | Middle Temporal Gyrus (R) | 56 | -10 | -16 | 0.003 |
|  |  | 62 | -2 | -24 | 0.003 |
| 7870 | Superior Temporal Gyrus (L) | -62 | -8 | -4 | 0.002 |
|  |  | -70 | -32 | 6 | 0.009 |
|  |  | -66 | -26 | 16 | 0.004 |
|  |  | -60 | -42 | 10 | 0.006 |
|  | Planum Temporale (L) | -64 | -12 | 6 | 0.003 |
|  | Temporal Pole (L) | -60 | 6 | -2 | 0.004 |
|  | Precentral Gyrus (L) | -60 | -2 | 12 | 0.005 |
|  |  | -42 | -14 | 38 | 0.007 |
|  |  | -62 | 8 | 20 | 0.009 |
|  | Middle Temporal Gyrus (L) | -64 | -8 | -16 | 0.005 |
|  |  | -68 | -26 | -2 | 0.010 |
|  | Supramarginal Gyrus (L) | -68 | -42 | 18 | 0.006 |
|  |  | -62 | -42 | 16 | 0.006 |
|  |  | -52 | -28 | 32 | 0.009 |
|  | Postcentral Gyrus (L) | -44 | -18 | 54 | 0.008 |
|  |  | -40 | -20 | 34 | 0.008 |
|  |  | -8 | -48 | 64 | 0.009 |
|  |  | -48 | -30 | 50 | 0.009 |
|  | Parietal Operculum Cortex (L) | -48 | -36 | 24 | 0.008 |
|  |  | -54 | -30 | 18 | 0.009 |
| 1159 | Cingulate Gyrus (L) | 0 | -2 | 44 | 0.012 |
|  |  | -6 | -16 | 44 | 0.032 |
|  |  | -2 | 10 | 36 | 0.032 |
|  |  | -10 | -16 | 40 | 0.049 |
|  | Cingulate Gyrus (R) | 4 | 12 | 38 | 0.032 |
|  |  | 14 | -14 | 36 | 0.049 |
|  |  | 4 | -10 | 42 | 0.019 |
|  |  | 10 | -20 | 42 | 0.023 |
|  | Juxtapositional Lobule Cortex (R) | 2 | -8 | 62 | 0.019 |
|  |  | 6 | -2 | 58 | 0.022 |
|  | Juxtapositional Lobule Cortex (L) | -8 | -10 | 66 | 0.024 |
|  | Precentral Gyrus (R) | 4 | -18 | 48 | 0.019 |
|  | Precentral Gyrus (L) | -2 | -22 | 52 | 0.031 |
| 410 | Amygdala (L) | -16 | -2 | -18 | 0.006 |
|  |  | -34 | -20 | -22 | 0.036 |
|  |  | -16 | -2 | -28 | 0.037 |
|  |  | -32 | -16 | -30 | 0.037 |
|  | Hippocampus (L) | -22 | -18 | -16 | 0.023 |
|  |  | -22 | -14 | -24 | 0.028 |
|  | Temporal Fusiform Cortex (L) | -36 | -24 | -30 | 0.036 |
| 56 | Inferior Temporal Gyrus (L) | -46 | -58 | -16 | 0.036 |
| 9 | Parahippocampal Gyrus (L) | -28 | -10 | -36 | 0.048 |
| *negative* | | | | | |
| 12 | Angular Gyrus (R) | 46 | -56 | 48 | 0.037 |
|  |  |  |  |  |  |
| ***EA Left ASD*** | | | | | |
| *positive* | | | | | |
| Cluster Size | Structure | x | y | z | p-value |
| 52623 | Temporal Pole (L) | -26 | 4 | -46 | 0.000 |
|  | Postcentral Gyrus (L) | -30 | -36 | 72 | 0.001 |
|  |  | -26 | -40 | 70 | 0.001 |
|  |  | -46 | -34 | 56 | 0.001 |
|  |  | -40 | -24 | 48 | 0.001 |
|  |  | -36 | -34 | 66 | 0.001 |
|  | Postcentral Gyrus (R) | 42 | -28 | 66 | 0.001 |
|  |  | 44 | -30 | 62 | 0.001 |
|  |  | 56 | -18 | 50 | 0.001 |
|  |  | 46 | -28 | 58 | 0.001 |
|  |  | 34 | -34 | 46 | 0.001 |
|  | Precentral Gyrus (L) | -40 | -22 | 62 | 0.001 |
|  |  | -42 | -14 | 56 | 0.001 |
|  |  | -54 | -10 | 44 | 0.001 |
|  | Precentral Gyrus (R) | 26 | -22 | 62 | 0.001 |
|  |  | 20 | -32 | 56 | 0.001 |
|  |  | 18 | -26 | 54 | 0.001 |
|  |  | 14 | -28 | 48 | 0.001 |
|  |  | 12 | -24 | 46 | 0.001 |
| *negative* | | | | | |
| 27 | Middle Frontal Gyrus (R) | 40 | 30 | 32 | 0.034 |
|  |  |  |  |  |  |
| ***EA Right ASD*** | | | | | |
| *positive* | | | | | |
| Cluster Size | Structure | x | y | z | p-value |
| 33517 | Temporal Pole (R) | 28 | 6 | -42 | 0.000 |
|  | Temporal Pole (L) | -48 | 8 | -10 | 0.001 |
|  | Central Opercular Cortex (R) | 46 | -16 | 12 | 0.001 |
|  | Superior Temporal Gyrus (L) | -66 | -18 | 10 | 0.001 |
|  |  | -58 | -2 | -12 | 0.001 |
|  |  | -50 | -14 | -12 | 0.001 |
|  |  | -56 | 2 | -14 | 0.001 |
|  | Superior Temporal Gyrus (R) | 56 | -30 | 0 | 0.001 |
|  |  | 58 | -26 | -2 | 0.001 |
|  |  | 68 | -26 | -2 | 0.001 |
|  |  | 66 | -20 | -4 | 0.001 |
|  | Planum Temporale (R) | 62 | -12 | 2 | 0.001 |
|  | Heschl's Gyrus (includes H1 and H2) (R) | 48 | -8 | 0 | 0.001 |
|  | Middle Temporal Gyrus (R) | 68 | -34 | 0 | 0.001 |
|  | Brain-Stem | -8 | -26 | -4 | 0.001 |
|  | Brain-Stem | 8 | -26 | -8 | 0.001 |
|  | Brain-Stem | 6 | -30 | -10 | 0.001 |
|  | Hippocampus (R) | 22 | -32 | -10 | 0.001 |
|  |  | 22 | -28 | -12 | 0.001 |

Cluster peaks and local maxima indicate positive and negative main effects with cortical areas from the (EA Left Controls) left entire amygdala in controls, (EA Right Controls) right entire amygdala in controls, (EA Left ASD) left entire amygdala in ASD and (EA Right ASD) right entire amygdala in ASD; (p < 0.05, FWE corrected).
