# Supplementary material for: Altered functional connectivity of the amygdaloid input nuclei in adolescents and young adults with autism spectrum disorder: a resting state fMRI study
Source: Mol Autism. 2016 Jan 28;7:13. doi: 10.1186/s13229-015-0060-x (PMC4730628; doi:10.1186/s13229-015-0060-x)
Supplement: Additional file 3: — Intrinsic superficial-cortical functional connectivity. Demonstrates main effects of left and right superficial-cortical partial correlation analysis in participants with Autism Spectrum Disorder and control subjects. (DOC 196 kb) [file 13229_2015_60_MOESM3_ESM.doc]

**Additional file 3. Intrinsic superficial-cortical functional connectivity.**

| ***SF Left Controls**** | | | | | |
| --- | --- | --- | --- | --- | --- |
| *positive* | | | | | |
| Cluster Size | Structure | x | y | z | p-value |
| 50762 | Parahippocampal Gyrus (L) | -14 | -6 | -26 | 0.000 |
|  |  | -14 | -10 | -24 | 0.001 |
|  | Precentral Gyrus (L) | -42 | -16 | 56 | 0.001 |
|  |  | -16 | -30 | 58 | 0.002 |
|  |  | -38 | -18 | 46 | 0.002 |
|  |  | -52 | -8 | 40 | 0.002 |
|  | Precentral Gyrus (R) | 4 | -30 | 52 | 0.004 |
|  |  | 42 | -12 | 46 | 0.002 |
|  | Postcentral Gyrus (R) | 38 | -32 | 64 | 0.002 |
|  |  | 30 | -34 | 62 | 0.002 |
|  |  | 44 | -18 | 56 | 0.002 |
|  |  | 6 | -38 | 58 | 0.004 |
|  |  | 42 | -18 | 48 | 0.002 |
|  | Postcentral Gyrus (L) | -24 | -30 | 56 | 0.002 |
|  |  | -50 | -18 | 40 | 0.002 |
|  |  | -58 | -22 | 36 | 0.002 |
|  |  | -60 | -16 | 32 | 0.002 |
|  | Superior Parietal Lobule (L) | -34 | -46 | 60 | 0.002 |
|  |  | -32 | -42 | 58 | 0.002 |
|  | Superior Parietal Lobule (R) | 40 | -38 | 56 | 0.002 |
| 9 | Frontal Medial Cortex (R) | 4 | 44 | -20 | 0.029 |
| 7 | Frontal Pole (L) | -2 | 62 | -6 | 0.031 |
|  |  |  |  |  |  |
| ***SF Right Controls*** | | | | | |
| *positive* | | | | | |
| Cluster Size | Structure | x | y | z | p-value |
| 2253 | Planum Polare (L) | -60 | -8 | 4 | 0.005 |
|  | Central Opercular Cortex (L) | -56 | -20 | 12 | 0.006 |
|  |  | -54 | -10 | 6 | 0.008 |
|  |  | -40 | 0 | 10 | 0.030 |
|  | Superior Temporal Gyrus (L) | -62 | 0 | -4 | 0.007 |
|  |  | -60 | -6 | -8 | 0.007 |
|  |  | -60 | -2 | -10 | 0.007 |
|  |  | -58 | 2 | -14 | 0.008 |
|  | Heschl's Gyrus (includes H1 and H2) (L) | -52 | -18 | 8 | 0.008 |
|  | Middle Temporal Gyrus | -58 | -8 | -12 | 0.008 |
|  | Middle Temporal Gyrus (L) | -56 | -50 | 10 | 0.024 |
|  |  | -56 | -46 | 8 | 0.024 |
|  | Insular Cortex (L) | -38 | -2 | -6 | 0.024 |
|  |  | -40 | 4 | 4 | 0.030 |
|  | Precentral Gyrus (L) | -44 | -10 | 56 | 0.033 |
|  | Postcentral Gyrus (L) | -50 | -18 | 34 | 0.025 |
|  |  | -42 | -20 | 36 | 0.033 |
|  |  | -42 | -14 | 32 | 0.033 |
|  | Supramarginal Gyrus (L) | -62 | -42 | 14 | 0.028 |
|  |  | -48 | -30 | 38 | 0.033 |
| 2933 | Hippocampus (R) | 20 | -6 | -22 | 0.000 |
|  | Central Opercular Cortex (R) | 60 | -8 | 6 | 0.013 |
|  |  | 42 | -16 | 12 | 0.028 |
|  |  | 54 | -16 | 10 | 0.041 |
|  | Superior Temporal Gyrus (R) | 60 | -8 | -8 | 0.013 |
|  |  | 66 | -18 | 8 | 0.015 |
|  |  | 52 | -30 | 2 | 0.032 |
|  |  | 58 | 2 | -14 | 0.036 |
|  | Middle Temporal Gyrus (R) | 64 | -6 | -12 | 0.015 |
|  | Precentral Gyrus (R) | 64 | -4 | 20 | 0.041 |
|  |  | 64 | 4 | 18 | 0.041 |
|  |  | 64 | 8 | 16 | 0.041 |
|  | Postcentral Gyrus (R) | 54 | -10 | 24 | 0.016 |
|  |  | 56 | -6 | 20 | 0.041 |
|  | Heschl's Gyrus (includes H1 and H2) (R) | 44 | -20 | 14 | 0.028 |
|  |  | 38 | -20 | 10 | 0.041 |
|  | Insular Cortex (R) | 36 | 6 | -16 | 0.033 |
|  |  | 40 | -6 | 6 | 0.036 |
|  | Planum Temporale (R) | 48 | -30 | 16 | 0.035 |
|  |  | 50 | -28 | 12 | 0.041 |
|  |  |  |  |  |  |
| ***SF Left ASD**** | | | | | |
| *positive* | | | | | |
| Cluster Size | Structure | x | y | z | p-value |
| 34867 | anterior Parahippocampal Gyrus (L) | -16 | -8 | -26 | 0.001 |
|  |  | -12 | -6 | -26 | 0.001 |
|  | anterior Parahippocampal Gyrus (R) | 12 | -4 | -22 | 0.001 |
|  |  | 18 | 2 | -26 | 0.001 |
|  | Planum Polare (R) | 50 | -6 | 0 | 0.001 |
|  | Middle Temporal Gyrus (L) | -46 | -40 | 0 | 0.001 |
|  |  | -62 | -36 | -4 | 0.001 |
|  | Superior Temporal Gyrus (R) | 58 | 2 | -6 | 0.001 |
|  | Insular Cortex (R) | 42 | 2 | -8 | 0.001 |
|  | Temporal Pole (R) | 46 | 10 | -16 | 0.001 |
|  | Hippocampus (R) | 14 | -6 | -18 | 0.001 |
|  |  | 22 | -8 | -20 | 0.001 |
|  | Brain-Stem | -4 | -10 | -18 | 0.001 |
|  | Hippocampus (L) | -22 | -18 | -18 | 0.001 |
|  | Temporal Pole (R) | 46 | 16 | -20 | 0.001 |
|  |  | 28 | 8 | -24 | 0.001 |
|  |  | 42 | 18 | -22 | 0.001 |
|  |  | 56 | 12 | -28 | 0.001 |
|  | Amygdala (R) | 20 | 0 | -20 | 0.001 |
|  |  | 28 | -20 | -20 | 0.001 |
|  |  |  |  |  |  |
| ***SF Right ASD**** | | | | | |
| *positive* | | | | | |
| Cluster Size | Structure | x | y | z | p-value |
| 47175 | Temporal Pole (R) | 26 | 14 | -36 | 0.000 |
|  | Superior Temporal Gyrus (L) | -60 | -36 | 4 | 0.001 |
|  |  | -52 | -38 | 4 | 0.001 |
|  | Superior Temporal Gyrus (R) | 68 | -12 | 2 | 0.001 |
|  | Postcentral Gyrus (R) | 62 | -12 | 40 | 0.001 |
|  |  | 42 | -12 | 32 | 0.001 |
|  | Precentral Gyrus (R) | 62 | -2 | 24 | 0.001 |
|  |  | 56 | -4 | 24 | 0.001 |
|  | Parietal Operculum Cortex (R) | 50 | -32 | 30 | 0.001 |
|  | Central Opercular Cortex (L) | -64 | -22 | 14 | 0.001 |
|  | Planum Temporale (R) | 58 | -28 | 12 | 0.001 |
|  |  | 62 | -24 | 10 | 0.001 |
|  | Planum Temporale (L) | -52 | -32 | 12 | 0.001 |
|  |  | -52 | -28 | 10 | 0.001 |
|  |  | -60 | -12 | 4 | 0.001 |
|  | Middle Temporal Gyrus (L) | -50 | -58 | 10 | 0.001 |
|  |  | -54 | -50 | 8 | 0.001 |
|  | Lateral Occipital Cortex (L) | -54 | -66 | 10 | 0.001 |
|  | Supramarginal Gyrus (R) | 62 | -38 | 6 | 0.001 |
|  | Central Opercular Cortex (L) | -54 | -4 | 4 | 0.001 |
|  | Heschl's Gyrus (includes H1 and H2) (R) | 48 | -10 | 4 | 0.001 |
|  |  |  |  |  |  |
| *negative* | | | | | |
| 5 | anterior Paracingulate Gyrus (L) | 0 | 32 | 36 | 0.047 |

Cluster peaks and local maxima indicate positive and negative main effects with cortical areas from the (SF Left Controls) left superficial amygdala in controls, (SF Right Controls) right superficial amygdala in controls, (SF Left ASD) left superficial amygdala in ASD and (SF Right ASD) right superficial amygdala in ASD; (p < 0.05, FWE corrected).

*Additional file 7 provides additional cluster information.
