# Supplementary material for: Altered functional connectivity of the amygdaloid input nuclei in adolescents and young adults with autism spectrum disorder: a resting state fMRI study
Source: Mol Autism. 2016 Jan 28;7:13. doi: 10.1186/s13229-015-0060-x (PMC4730628; doi:10.1186/s13229-015-0060-x)
Supplement: Additional file 4: — Intrinsic laterobasal-cortical functional connectivity. Demonstrates main effects of left and right laterobasal-cortical partial correlation analysis in participants with autism spectrum disorder and control subjects. (DOCX 384 kb) [file 13229_2015_60_MOESM4_ESM.docx]

**Additional file 4. Intrinsic laterobasal-cortical functional connectivity.**

| ***LB Left Controls*** | | | | | |
| --- | --- | --- | --- | --- | --- |
| *positive* | | | | | |
| Cluster Size | Structure | x | y | z | p-value |
| 4608 | Parahippocampal Gyrus (L) | -26 | -8 | -34 | 0.000 |
|  | Temporal Pole (L) | -54 | 4 | -28 | 0.005 |
|  |  | -38 | 6 | -30 | 0.005 |
|  |  | -44 | 4 | -32 | 0.005 |
|  |  | -58 | 6 | -14 | 0.012 |
|  | Temporal Occipital Fusiform Cortex (L) | -36 | -52 | -20 | 0.014 |
|  | Planum Temporale (L) | -60 | -24 | 12 | 0.015 |
|  |  | -64 | -26 | 12 | 0.015 |
|  |  | -54 | -34 | 16 | 0.018 |
|  | Lateral Occipital Cortex (L) | -52 | -68 | 10 | 0.015 |
|  | Frontal Orbital Cortex (L) | -34 | 18 | -26 | 0.015 |
|  | Temporal Fusiform Cortex (L) | -36 | -34 | -28 | 0.015 |
|  | Heschl's Gyrus (includes H1 and H2) (L) | -50 | -22 | 10 | 0.017 |
|  | Temporal Fusiform Cortex (L) | -36 | -14 | -36 | 0.017 |
|  | Middle Temporal Gyrus (L) | -64 | -10 | -18 | 0.018 |
|  |  | -64 | -16 | -20 | 0.018 |
|  | Superior Temporal Gyrus (L) | -64 | -38 | 18 | 0.019 |
|  | Postcentral Gyrus (R) | 60 | -14 | 48 | 0.012 |
|  |  | 68 | -14 | 22 | 0.014 |
|  |  | 56 | -10 | 44 | 0.017 |
| 2645 | Precentral Gyrus (R) | 42 | -14 | 52 | 0.017 |
|  | Superior Temporal Gyrus (R) | 68 | -28 | 18 | 0.017 |
|  |  | 70 | -24 | 10 | 0.017 |
|  |  | 62 | -34 | 12 | 0.025 |
|  | Planum Temporale (R) | 64 | -30 | 18 | 0.017 |
|  | Temporal Pole (R) | 54 | 18 | -16 | 0.020 |
|  | Postcentral Gyrus (R) | 66 | -12 | 32 | 0.021 |
|  | Insular Cortex (R) | 40 | -14 | -2 | 0.022 |
|  |  | 36 | -20 | 16 | 0.028 |
|  | Planum Polare (R) | 48 | -2 | -8 | 0.024 |
|  |  | 66 | -4 | 4 | 0.028 |
|  |  | 66 | -6 | -4 | 0.028 |
|  | Parietal Operculum Cortex (R) | 56 | -32 | 20 | 0.026 |
|  | Planum Temporale (R) | 40 | -32 | 16 | 0.027 |
|  | Central Opercular Cortex (R) | 50 | -18 | 14 | 0.027 |
|  | Precentral Gyrus (R) | 38 | -8 | 66 | 0.028 |
|  | Heschl's Gyrus (includes H1 and H2) (R) | 48 | -22 | 12 | 0.028 |
| 2419 | Lateral Occipital Cortex (R) | 30 | -84 | 28 | 0.021 |
|  |  | 44 | -76 | -16 | 0.023 |
|  |  | 38 | -76 | -18 | 0.023 |
|  |  | 56 | -64 | -6 | 0.025 |
|  |  | 42 | -68 | 0 | 0.028 |
|  | Occipital Fusiform Gyrus (R) | 28 | -66 | -8 | 0.028 |
|  |  | 24 | -66 | -10 | 0.028 |
|  |  | 24 | -74 | -6 | 0.033 |
|  | Temporal Occipital Fusiform Cortex (R) | 44 | -46 | -18 | 0.030 |
|  | Lateral Occipital Cortex (R) | 32 | -74 | 16 | 0.031 |
|  |  | 44 | -86 | 2 | 0.033 |
|  |  | 42 | -74 | 0 | 0.033 |
|  |  | 44 | -66 | -4 | 0.033 |
|  | Lingual Gyrus (R) | 12 | -74 | 2 | 0.031 |
|  |  | 14 | -74 | -4 | 0.033 |
|  | Occipital Pole (R) | 24 | -90 | 26 | 0.033 |
|  |  | 16 | -96 | 20 | 0.033 |
|  |  | 36 | -96 | 4 | 0.033 |
| 1226 | Precentral Gyrus (L) | -44 | -14 | 56 | 0.014 |
|  |  | -38 | -12 | 56 | 0.015 |
|  |  | -40 | -22 | 60 | 0.024 |
|  |  | -38 | -24 | 56 | 0.049 |
|  |  | -28 | -24 | 54 | 0.049 |
|  | Postcentral Gyrus (L) | -42 | -18 | 48 | 0.024 |
|  |  | -28 | -38 | 70 | 0.041 |
|  |  | -52 | -28 | 52 | 0.044 |
|  |  | -54 | -24 | 40 | 0.047 |
|  |  | -30 | -32 | 66 | 0.049 |
|  | Superior Parietal Lobule (L) | -32 | -42 | 54 | 0.042 |
|  |  | -32 | -40 | 50 | 0.049 |
|  | Supramarginal Gyrus (L) | -52 | -26 | 32 | 0.049 |
| 1212 | Lateral Occipital Cortex (L) | -20 | -80 | 42 | 0.020 |
|  |  | -26 | -60 | 50 | 0.026 |
|  |  | -26 | -80 | 24 | 0.026 |
|  |  | -22 | -88 | 24 | 0.026 |
|  |  | -18 | -62 | 46 | 0.049 |
|  | Supracalcarine Cortex (L) | -10 | -66 | 14 | 0.047 |
|  | Superior Parietal Lobule (L) | -16 | -56 | 58 | 0.049 |
|  | Cuneal Cortex (L) | -12 | -76 | 20 | 0.049 |
|  |  | -4 | -80 | 18 | 0.049 |
|  |  | -18 | -72 | 16 | 0.049 |
|  | Occipital Pole (L) | -4 | -94 | 20 | 0.049 |
|  |  | -24 | -90 | 16 | 0.049 |
|  | Intracalcarine Cortex (L) | 0 | -70 | 10 | 0.049 |
| 73 | Parahippocampal Gyrus (R) | 32 | -18 | -30 | 0.047 |
|  | Temporal Fusiform Cortex (R) | 40 | -18 | -28 | 0.048 |
|  |  |  |  |  |  |
| ***LB Right Controls*** | | | | | |
| *positive* | | | | | |
| Cluster Size | Structure | x | y | z | p-value |
| 3852 | Temporal Fusiform Cortex, anterior (R) | 32 | -6 | -36 | 0.000 |
|  | Temporal Pole (R) | 40 | 14 | -38 | 0.006 |
|  |  | 32 | 10 | -38 | 0.006 |
|  |  | 40 | 10 | -38 | 0.006 |
|  |  | 32 | 10 | -42 | 0.006 |
|  |  | 58 | 12 | -14 | 0.010 |
|  |  | 54 | 6 | -18 | 0.010 |
|  | Hippocampus (R) | 26 | -20 | -22 | 0.008 |
|  | Temporal Fusiform Cortex,posterior (R) | 42 | -16 | -30 | 0.008 |
|  | Planum Temporale (R) | 62 | -8 | 4 | 0.009 |
|  |  | 60 | -20 | 10 | 0.010 |
|  |  | 60 | -16 | 6 | 0.010 |
|  | Middle Temporal Gyrus,anterior (R) | 60 | -4 | -22 | 0.009 |
|  | Middle Temporal Gyrus,posterior (R) | 60 | -14 | -20 | 0.010 |
|  | Middle Temporal Gyrus,posterior (R) | 58 | -8 | -26 | 0.010 |
|  | Parietal Operculum Cortex (R) | 56 | -28 | 20 | 0.010 |
|  | Superior Temporal Gyrus,posterior (R) | 64 | -32 | 12 | 0.010 |
|  | Superior Temporal Gyrus,anterior (R) | 60 | 2 | -10 | 0.010 |
|  |  | 58 | 4 | -16 | 0.010 |
|  | Planum Polare (R) | 62 | 4 | 2 | 0.010 |
| 225 | Postcentral Gyrus (L) | -10 | -48 | 64 | 0.027 |
|  |  | -22 | -42 | 58 | 0.034 |
|  | Superior Parietal Lobule (L) | -38 | -40 | 60 | 0.037 |
|  | Postcentral Gyrus (L) | -22 | -40 | 54 | 0.037 |
| 207 | Cingulate Gyrus,anterior (R) | 6 | -12 | 44 | 0.031 |
|  | Cingulate Gyrus,anterior (L) | 0 | -2 | 44 | 0.033 |
|  |  | -4 | -14 | 44 | 0.048 |
|  | Precentral Gyrus (L) | 0 | -22 | 50 | 0.048 |
| 207 | Postcentral Gyrus (R) | 56 | -10 | 48 | 0.025 |
|  |  | 62 | -6 | 36 | 0.033 |
|  |  | 62 | -6 | 32 | 0.042 |
|  | Precentral Gyrus (R) | 58 | -4 | 44 | 0.028 |
|  |  | 52 | -8 | 34 | 0.042 |
| 153 | Superior Temporal Gyrus,anterior (L) | -64 | -8 | -4 | 0.018 |
|  | Planum Temporale (L) | -64 | -14 | 6 | 0.031 |
|  | Temporal Pole (L) | -60 | 6 | -4 | 0.045 |
| 54 | Postcentral Gyrus (L) | -44 | -18 | 54 | 0.046 |
| 47 | Planum Temporale (R) | 40 | -28 | 14 | 0.045 |
| 42 | Juxtapositional Lobule Cortex (R) | 2 | -8 | 62 | 0.033 |
|  |  | 2 | -8 | 62 | 0.034 |
|  |  | 6 | 0 | 60 | 0.041 |
| 24 | Amygdala (L) | -16 | -2 | -18 | 0.023 |
| 23 | Frontal Orbital Cortex (R) | 44 | 28 | -18 | 0.048 |
| 18 | Parietal Operculum Cortex (L) | -46 | -36 | 22 | 0.050 |
| 18 | Precentral Gyrus (R) | 52 | 4 | 14 | 0.049 |
| 17 | Supramarginal Gyrus, posterior (L) | -64 | -42 | 16 | 0.050 |
|  |  |  |  |  |  |
| ***LB Left ASD*** | | | | | |
| *positive* | | | | | |
| Cluster Size | Structure | x | y | z | p-value |
| 7681 | Temporal Fusiform Cortex (L) | -22 | -2 | -44 | 0.000 |
|  |  | -30 | -26 | -30 | 0.001 |
|  |  | -36 | -40 | -30 | 0.001 |
|  |  | -38 | -18 | -34 | 0.001 |
|  |  | -42 | -20 | -34 | 0.001 |
|  | Superior Temporal Gyrus (L) | -56 | -6 | -8 | 0.001 |
|  | Middle Temporal Gyrus (L) | -54 | -16 | -12 | 0.001 |
|  |  | -52 | -2 | -22 | 0.001 |
|  | Temporal Pole (L) | -48 | 16 | -28 | 0.001 |
|  |  | -46 | 8 | -28 | 0.001 |
|  |  | -46 | 20 | -30 | 0.001 |
|  |  | -24 | 14 | -42 | 0.001 |
|  | Cerebellum (L) | -16 | -40 | -28 | 0.001 |
|  |  | -28 | -42 | -32 | 0.001 |
|  | Parahippocampal Gyrus (L) | -24 | -12 | -40 | 0.001 |
|  | Frontal Orbital Cortex (L) | -30 | 32 | -16 | 0.004 |
|  | Brain Stem | -22 | -36 | -30 | 0.001 |
| 5630 | Temporal Pole (R) | 48 | 8 | -30 | 0.002 |
|  |  | 48 | 8 | -30 | 0.003 |
|  |  | 26 | 10 | -40 | 0.005 |
|  | Planum Polare (R) | 54 | 4 | -6 | 0.003 |
|  | Superior Temporal Gyrus (R) | 66 | -4 | -8 | 0.004 |
|  |  | 60 | -6 | -8 | 0.004 |
|  |  | 58 | -6 | -12 | 0.004 |
|  |  | 60 | 2 | -14 | 0.004 |
|  | Inferior Temporal Gyrus (R) | 44 | -28 | -22 | 0.004 |
|  | Inferior Temporal Gyrus (R) | 46 | 0 | -36 | 0.004 |
|  | Temporal Fusiform Cortex (R) | 40 | -18 | -24 | 0.004 |
|  |  | 38 | -10 | -28 | 0.004 |
|  |  | 42 | -38 | -24 | 0.005 |
|  |  | 40 | -16 | -28 | 0.005 |
|  | Temporal Occipital Fusiform Cortex (R) | 46 | -48 | -22 | 0.005 |
|  | Parahippocampal Gyrus (R) | 26 | -20 | -22 | 0.004 |
|  | Hippocampus (R) | 32 | -6 | -24 | 0.004 |
|  |  | 26 | -12 | -24 | 0.004 |
|  |  | 28 | -6 | -26 | 0.004 |
|  |  | 32 | -24 | -10 | 0.006 |
|  | Brain Stem | 16 | -36 | -28 | 0.005 |
| 718 | Precentral Gyrus (R) | 30 | -28 | 52 | 0.026 |
|  |  | 30 | -28 | 52 | 0.026 |
|  |  | 36 | -16 | 40 | 0.028 |
|  |  | 44 | -6 | 36 | 0.042 |
|  |  | 38 | -16 | 36 | 0.046 |
|  | Postcentral Gyrus (R) | 34 | -26 | 50 | 0.028 |
|  |  | 42 | -22 | 44 | 0.028 |
|  |  | 56 | -12 | 48 | 0.038 |
|  |  | 36 | -32 | 50 | 0.046 |
|  |  | 56 | -16 | 40 | 0.046 |
| 23 | Planum Temporale (R) | 64 | -18 | 8 | 0.043 |
|  | Superior Temporal Gyrus (R) | 68 | -24 | 10 | 0.043 |
| 22 | Lateral Occipital Cortex (L) | -36 | -68 | 0 | 0.045 |
| 8 | Superior Temporal Gyrus (R) | 68 | -28 | 18 | 0.047 |
|  |  |  |  |  |  |
| ***LB Right ASD*** | | | | | |
| *positive* | | | | | |
| Cluster Size | Structure | x | y | z | p-value |
| 10471 | Temporal Fusiform Cortex (R) | 30 | -4 | -38 | 0.000 |
|  |  | 38 | -26 | -26 | 0.001 |
|  | Temporal Fusiform Cortex (L) | -42 | -22 | -26 | 0.001 |
|  | Hippocampus (R) | 32 | -20 | -10 | 0.001 |
|  | Middle Temporal Gyrus (L) | -54 | -20 | -20 | 0.001 |
|  |  | -60 | -14 | -22 | 0.001 |
|  |  | -64 | -10 | -26 | 0.001 |
|  | Middle Temporal Gyrus (R) | 66 | -12 | -24 | 0.001 |
|  |  | 58 | -8 | -26 | 0.001 |
|  | Temporal Pole (R) | 36 | 16 | -24 | 0.001 |
|  | Temporal Pole (L) | -40 | 6 | -24 | 0.001 |
|  | Inferior Temporal Gyrus (R) | 60 | -26 | -24 | 0.001 |
|  |  | 60 | -22 | -26 | 0.001 |
|  |  | 48 | -28 | -24 | 0.001 |
|  | Parahippocampal Gyrus (L) | -18 | -12 | -28 | 0.001 |
|  |  | -18 | -2 | -30 | 0.001 |
|  |  | -26 | -16 | -30 | 0.001 |
|  | Parahippocampal Gyrus (R) | 26 | -16 | -30 | 0.001 |
|  | Frontal Orbital Cortex (R) | 28 | 12 | -24 | 0.001 |
| 251 | Frontal Pole (L) | -38 | 36 | -20 | 0.035 |
|  |  | -38 | 36 | -20 | 0.035 |
|  | Frontal Orbital Cortex (L) | -38 | 32 | -20 | 0.037 |
|  |  | -30 | 30 | -20 | 0.039 |
|  |  | -46 | 28 | -16 | 0.041 |
|  |  | -42 | 28 | -18 | 0.042 |
|  |  | -26 | 26 | -20 | 0.046 |
| 136 | Occipital Pole (R) | 24 | -92 | 6 | 0.026 |
|  |  | 24 | -92 | 6 | 0.026 |
|  |  | 18 | -86 | 10 | 0.030 |
|  |  | 24 | -90 | 0 | 0.032 |
|  | Lateral Occipital Cortex (R) | 28 | -88 | -2 | 0.041 |
| 125 | Middle Temporal Gyrus (L) | -58 | -42 | -10 | 0.041 |
|  |  | -58 | -42 | -10 | 0.041 |
|  |  | -62 | -32 | -4 | 0.047 |
|  | Inferior Temporal Gyrus (L) | -58 | -44 | -18 | 0.043 |
| 119 | Frontal Pole (R) | 40 | 36 | -20 | 0.041 |
|  |  | 40 | 36 | -20 | 0.042 |
|  |  | 46 | 42 | -20 | 0.047 |
|  | Frontal Orbital Cortex (R) | 30 | 32 | -16 | 0.042 |
|  |  | 38 | 24 | -18 | 0.047 |
| 17 | Frontal Pole (R) | 28 | 54 | -16 | 0.047 |
|  |  | 28 | 54 | -16 | 0.047 |
|  |  | 28 | 48 | -16 | 0.048 |
| 12 | Cerebellum (L) | -18 | -50 | -20 | 0.047 |
| 9 | Lingual Gyrus (R) | 22 | -74 | -2 | 0.050 |
|  | Amygdala (L) | -26 | -2 | -30 | 0.001 |

Cluster peaks and local maxima indicate positive and negative main effects with cortical areas from the (LB Left Controls) left laterobasal amygdala in controls, (LB Right Controls) right laterobasal amygdala in controls, (LB Left ASD) left laterobasal amygdala in ASD and (LB Right ASD) right laterobasal amygdala in ASD; (p < 0.05, FWE corrected).
