# Supplementary material for: Altered functional connectivity of the amygdaloid input nuclei in adolescents and young adults with autism spectrum disorder: a resting state fMRI study
Source: Mol Autism. 2016 Jan 28;7:13. doi: 10.1186/s13229-015-0060-x (PMC4730628; doi:10.1186/s13229-015-0060-x)
Supplement: Additional file 5: — Intrinsic centromedial-cortical functional connectivity. Demonstrates main effects of left and right centromedial-cortical partial correlation analysis in participants with autism spectrum disorder and control subjects. (DOCX 109 kb) [file 13229_2015_60_MOESM5_ESM.docx]

**Additional file 5. Intrinsic centromedial-cortical functional connectivity.**

| ***CM Right Controls*** | | | | | |
| --- | --- | --- | --- | --- | --- |
| *negative* | | | | | |
| Cluster Size | Structure | x | y | z | p-value |
| 15 | Occipital Fusiform Gyrus (R) | 28 | -64 | -10 | 0.040 |
|  |  |  |  |  |  |
| ***CM Left ASD*** | | | | | |
| *positive* | | | | | |
| Cluster Size | Structure | x | y | z | p-value |
| 1592 | Postcentral Gyrus (L) | -36 | -22 | 40 | 0.004 |
|  |  | -6 | -36 | 56 | 0.014 |
|  |  | -28 | -28 | 48 | 0.016 |
|  |  | -42 | -14 | 30 | 0.016 |
|  | Precentral Gyrus (L) | -18 | -26 | 66 | 0.007 |
|  |  | -24 | -24 | 64 | 0.007 |
|  |  | -52 | -16 | 44 | 0.007 |
|  |  | -10 | -34 | 62 | 0.009 |
|  |  | -46 | -12 | 50 | 0.009 |
|  | Precentral Gyrus (R) | 2 | -24 | 54 | 0.029 |
|  |  | 6 | -20 | 62 | 0.033 |
|  |  | 12 | -28 | 62 | 0.035 |
|  | Postcentral Gyrus (R) | 10 | -34 | 64 | 0.033 |
| 244 | Amygdala (L) | -22 | -8 | -16 | 0.000 |
|  | Insular Cortex (L) | -34 | -12 | -6 | 0.049 |
| 73 | Precentral Gyrus (R) | 40 | -14 | 60 | 0.007 |
|  | Postcentral Gyrus (R) | 44 | -24 | 58 | 0.037 |
| 6 | Precentral Gyrus (R) | 42 | -10 | 40 | 0.046 |
|  |  |  |  |  |  |
| ***CM Right ASD*** | | | | | |
| *positive* |  |  |  |  |  |
| Cluster Size | Structure | x | y | z | p-value |
| 3094 | Amygdala (R) | 30 | -8 | -14 | 0.000 |
|  | Thalamus (L) | -2 | -10 | 6 | 0.006 |
|  |  | -2 | -24 | 6 | 0.010 |
|  | Thalamus (R) | 12 | -14 | 4 | 0.007 |
|  |  | 4 | -24 | 6 | 0.010 |
|  |  | 6 | -4 | 2 | 0.029 |
|  |  | 6 | 0 | 0 | 0.029 |
|  |  | 24 | -14 | 18 | 0.024 |
|  | Pallidum (R) | 18 | 2 | 4 | 0.008 |
|  |  | 22 | 18 | 2 | 0.028 |
|  | Central Opercular Cortex (R) | 48 | -4 | 10 | 0.004 |
|  |  | 52 | -14 | 12 | 0.007 |
|  |  | 42 | -2 | 14 | 0.014 |
|  | Postcentral Gyrus (R) | 50 | -6 | 22 | 0.006 |
|  | Precentral Gyrus (R) | 62 | 10 | 14 | 0.028 |
|  | Heschl's Gyrus (includes H1 and H2) (R) | 52 | -12 | 6 | 0.007 |
|  |  | 54 | -16 | 4 | 0.015 |
|  | Supramarginal Gyrus (R) | 56 | -22 | 26 | 0.027 |
|  |  | 64 | 8 | 24 | 0.032 |
|  | Frontal Operculum Cortex (R) | 40 | 12 | 4 | 0.032 |
| 2 | Putamen (L) | -24 | 0 | -10 | 0.046 |
|  |  |  |  |  |  |
| *negative* | | | | | |
| 268 | Lateral Occipital Cortex, superior (R) | 26 | -72 | 40 | 0.022 |
|  |  | 28 | -78 | 38 | 0.023 |
|  |  | 28 | -82 | 46 | 0.028 |
|  |  | 28 | -68 | 28 | 0.030 |
|  |  | 28 | -72 | 50 | 0.039 |
| 161 | Lateral Occipital Cortex, superior (L) | -12 | -78 | 44 | 0.013 |
|  |  | -10 | -86 | 42 | 0.028 |
|  |  | -26 | -88 | 40 | 0.028 |
| 38 | Parietal Lobule, superior (R) | 28 | -54 | 38 | 0.033 |

Cluster peaks and local maxima indicate positive and negative main effects with cortical areas from the (CM Right Controls) right centromedial amygdala in controls, (CM Left ASD) left centromedial amygdala in ASD and (CM Right ASD) right centromedial amygdala in ASD; (p < 0.05, FWE corrected).
