# Supplementary material for: Altered functional connectivity of the amygdaloid input nuclei in adolescents and young adults with autism spectrum disorder: a resting state fMRI study
Source: Mol Autism. 2016 Jan 28;7:13. doi: 10.1186/s13229-015-0060-x (PMC4730628; doi:10.1186/s13229-015-0060-x)
Supplement: Additional file 6: — Intrinsic amygdalo-cortical functional connectivity in superficial subregion. Demonstrates a selection of superficial-cortical partial correlation main effects with adjusted significant thresholds in participants with autism spectrum disorder and control subjects. (DOC 253 kb) [file 13229_2015_60_MOESM6_ESM.doc]

**Additional file 6. Intrinsic amygdalo-cortical functional connectivity in superficial subregion.**

| ***SF Left Controls*** | | | | | |
| --- | --- | --- | --- | --- | --- |
| *positive* | | | | | |
| Cluster Size | Structure | x | y | z | p-value |
| 883 | Precentral Gyrus (L) | -42 | -16 | 56 | 0.001 |
| 6 | Precentral Gyrus (L) | -2 | -30 | 70 | 0.001 |
| 5 | Precentral Gyrus (R) | 64 | 2 | 20 | 0.001 |
| 608 | Postcentral Gyrus (R) | 38 | -32 | 64 | 0.001 |
| 6 | Postcentral Gyrus (L) | -44 | -28 | 44 | 0.001 |
| 3 | Postcentral Gyrus (L) | -58 | -24 | 52 | 0.001 |
| 1 | Postcentral Gyrus (L) | -64 | -6 | 30 | 0.001 |
| 480 | Parahippocampal Gyrus, anterior division (R) | -14 | -6 | -26 | 0.001 |
| 3 | Parahippocampal Gyrus, posterior division (L) | -26 | -30 | -22 | 0.001 |
| 311 | Cingulate Gyrus, posterior division (R) | 4 | -20 | 48 | 0.001 |
| 24 | Cingulate Gyrus (R) | 6 | -46 | 30 | 0.001 |
| 289 | Temporal Pole (R) | 52 | 16 | -30 | 0.001 |
| 170 | Superior Temporal Gyrus, posterior division (L) | -60 | -18 | 0 | 0.001 |
| 137 | Lateral Occipital Cortex, inferior division (L) | -50 | -72 | -6 | 0.001 |
| 94 | Postcentral Gyrus (R) | 40 | -14 | 34 | 0.001 |
| 78 | Occipital Fusiform Gyrus (R) | 30 | -84 | -18 | 0.001 |
| 67 | Right Amygdala (R) | 26 | -6 | -22 | 0.001 |
| 61 | Superior Parietal Lobule (R) | 26 | -56 | 56 | 0.001 |
| 55 | Superior Parietal Lobule (L) | -30 | -56 | 56 | 0.001 |
| 1 | Superior Parietal Lobule (R) | 36 | -52 | 64 | 0.001 |
| 36 | Superior Temporal Gyrus, posterior division (L) | -60 | -8 | -8 | 0.001 |
| 34 | Inferior Temporal Gyrus (R) | 50 | -42 | -18 | 0.001 |
| 32 | Middle Temporal Gyrus (L) | -56 | -4 | -20 | 0.001 |
| 17 | Middle Temporal Gyrus, temporooccipital part (R) | 60 | -44 | 12 | 0.001 |
| 11 | Middle Temporal Gyrus, temporooccipital part (L) | -48 | -48 | 4 | 0.001 |
| 10 | Middle Temporal Gyrus, temporooccipital part (L) | -62 | -46 | 6 | 0.001 |
| 30 | Temporal Occipital Fusiform Cortex (L) | -44 | -54 | -18 | 0.001 |
| 7 | Temporal Occipital Fusiform Cortex (L) | -30 | -48 | -20 | 0.001 |
| 24 | Precuneous Cortex (L) | -2 | -54 | 16 | 0.001 |
| 18 | Lingual Gyrus (R) | 20 | -38 | -12 | 0.001 |
| 8 | Lingual Gyrus (L) | -12 | -50 | -8 | 0.001 |
| 1 | Lingual Gyrus (L) | -6 | -46 | -6 | 0.001 |
| 1 | Lingual Gyrus (L) | -24 | -46 | -10 | 0.001 |
| 13 | Temporal Pole (R) | 42 | 18 | -38 | 0.001 |
| 12 | Insular Cortex (R) | 36 | -8 | 2 | 0.001 |
| 11 | Frontal Orbital Cortex (R) | 40 | 32 | -12 | 0.001 |
| 5 | Temporal Fusiform Cortex, posterior division (L) | -40 | -44 | -26 | 0.001 |
| 2 | Temporal Fusiform Cortex, posterior division (R) | 42 | -14 | -28 | 0.001 |
| 4 | Precuneous Cortex (R) | 16 | -56 | 8 | 0.001 |
| 4 | Inferior Frontal Gyrus, pars triangularis (L) | -54 | 26 | 6 | 0.001 |
| 4 | Precuneous Cortex (L) | -18 | -58 | 10 | 0.001 |
| 4 | Lateral Occipital Cortex, inferior division (L) | -42 | -74 | -12 | 0.001 |
| 3 | Lateral Occipital Cortex, inferior division (R) | 30 | -86 | 8 | 0.001 |
| 1 | Lateral Occipital Cortex, superior division (R) | 56 | -62 | 18 | 0.001 |
| 4 | Occipital Fusiform Gyrus (L) | -14 | -88 | -14 | 0.001 |
| 4 | Occipital Fusiform Gyrus (L) | -28 | -82 | -16 | 0.001 |
| 2 | Precuneous Cortex (R) | 12 | -60 | 18 | 0.001 |
| 2 | Subcallosal Cortex (R) | 2 | 8 | -12 | 0.001 |
| 1 | Hippocampus (R) | 32 | -22 | -16 | 0.001 |
|  |  |  |  |  |  |
| ***SF Left ASD*** | | | | | |
| *positive* | | | | | |
| Cluster Size | Structure | x | y | z | p-value |
| 650 | Parahippocampal Gyrus, anterior division (L) | -16 | -8 | -26 | 0.000 |
| 34 | Parahippocampal Gyrus, posterior division (L) | -10 | -36 | -8 | 0.003 |
| 20 | Parahippocampal Gyrus, posterior division (L) | -14 | -30 | -12 | 0.003 |
| 429 | Amygdala (R) | 20 | 0 | -20 | 0.000 |
| 220 | Insular Cortex (R) | 42 | 2 | -8 | 0.001 |
| 127 | Middle Temporal Gyrus, posterior division (L) | -62 | -36 | -4 | 0.001 |
| 44 | Middle Temporal Gyrus, anterior division (L) | -60 | -6 | -18 | 0.003 |
| 17 | Middle Temporal Gyrus, anterior division (R) | 64 | 0 | -22 | 0.003 |
| 4 | Middle Temporal Gyrus, posterior division (R) | 66 | -34 | -2 | 0.005 |
| 115 | Temporal Pole (R) | 56 | 12 | -28 | 0.001 |
| 10 | Temporal Pole (R) | 52 | 4 | -40 | 0.004 |
| 4 | Temporal Pole (R) | 36 | 18 | -44 | 0.005 |
| 95 | Frontal Operculum Cortex (L) | -46 | 28 | 0 | 0.001 |
| 55 | Central Opercular Cortex (R) | 62 | -2 | 6 | 0.002 |
| 1 | Central Opercular Cortex (L) | -54 | -14 | 12 | 0.006 |
| 48 | Precentral Gyrus (L) | 0 | -20 | 58 | 0.002 |
| 43 | Precentral Gyrus (L) | -40 | -16 | 52 | 0.002 |
| 41 | Precentral Gyrus (L) | -54 | -10 | 44 | 0.003 |
| 40 | Precentral Gyrus (L) | -18 | -28 | 58 | 0.003 |
| 6 | Precentral Gyrus (L) | -48 | 8 | 28 | 0.005 |
| 4 | Precentral Gyrus (L) | -64 | -4 | 12 | 0.005 |
| 3 | Precentral Gyrus (L) | -4 | -32 | 70 | 0.005 |
| 1 | Precentral Gyrus (L) | -32 | -24 | 52 | 0.006 |
| 45 | Precentral Gyrus (R) | 22 | -28 | 56 | 0.002 |
| 1 | Precentral Gyrus (R) | 30 | -22 | 54 | 0.006 |
| 26 | Frontal Orbital Cortex (L) | -28 | 12 | -22 | 0.003 |
| 1 | Frontal Orbital Cortex (R) | 26 | 28 | -8 | 0.006 |
| 1 | Frontal Orbital Cortex (R) | 26 | 24 | -10 | 0.006 |
| 20 | Hippocampus (R) | 28 | -30 | -14 | 0.003 |
| 18 | Planum Polare (R) | 40 | 2 | -20 | 0.003 |
| 17 | Superior Temporal Gyrus, anterior division (L) | -60 | -2 | 0 | 0.003 |
| 15 | Superior Temporal Gyrus, anterior division (L) | -56 | 2 | -14 | 0.003 |
| 13 | Brain-Stem | 10 | -34 | -4 | 0.003 |
| 11 | Inferior Temporal Gyrus, temporooccipital division (L) | -48 | -52 | -14 | 0.004 |
| 9 | Thalamus (L) | -2 | 2 | -2 | 0.004 |
| 7 | Angular Gyrus (Supramarginal Gyrus, posterior division) (L) | -60 | -50 | 14 | 0.004 |
| 2 | Supramarginal Gyrus, posterior division (L) | -66 | -48 | 22 | 0.006 |
| 7 | Caudate (R) | 10 | 18 | 6 | 0.004 |
| 1 | Caudate (L) | -4 | 12 | 4 | 0.006 |
| 7 | Temporal Pole (R) | 42 | 22 | -32 | 0.004 |
| 7 | Postcentral Gyrus (R) | 60 | -8 | 20 | 0.004 |
| 7 | Postcentral Gyrus (L) | -66 | -8 | 28 | 0.005 |
| 6 | Postcentral Gyrus (L) | -42 | -24 | 50 | 0.005 |
| 2 | Postcentral Gyrus (L) | -66 | -14 | 32 | 0.006 |
| 4 | Postcentral Gyrus (L) | -50 | -26 | 52 | 0.005 |
| 2 | Postcentral Gyrus (R) | 66 | -14 | 30 | 0.006 |
| 2 | Postcentral Gyrus (R) | 54 | -10 | 28 | 0.006 |
| 2 | Planum Temporale (L) | -60 | -30 | 10 | 0.006 |
| 1 | Juxtapositional Lobule Cortex (L) | -2 | -6 | 56 | 0.006 |
| 1 | Superior Temporal Gyrus, posterior division (L) | -54 | -20 | -2 | 0.006 |
| 1 | Superior Temporal Gyrus, posterior division (L) | -52 | -20 | -6 | 0.006 |
|  |  |  |  |  |  |
| ***SF Right ASD*** | | | | | |
| *positive* | | | | | |
| Cluster Size | Structure | x | y | z | p-value |
| 563 | Frontal Medial Cortex (R) | 2 | 54 | -12 | 0.010 |
| 528 | Postcentral Gyrus (L) | -50 | -16 | 34 | 0.013 |
| 193 | Postcentral Gyrus (L) | 0 | -36 | 70 | 0.029 |
| 182 | Central Opercular Cortex (L) | -46 | -18 | 12 | 0.027 |
| 175 | Cingulate Gyrus, anterior division (R) | 2 | -8 | 44 | 0.029 |
| 15 | Cingulate Gyrus, anterior division (L) | -2 | 36 | 2 | 0.044 |
| 10 | Cingulate Gyrus, posterior division (L) | -14 | -40 | 2 | 0.045 |
| 172 | Cerebellum, right VI (R) | 26 | -68 | -24 | 0.032 |
| 17 | Cerebellum, right IX (R) | 12 | -48 | -36 | 0.044 |
| 164 | Cerebellum, left VI (L) | -16 | -64 | -22 | 0.027 |
| 12 | Cerebellum, left IX (L) | -8 | -56 | -40 | 0.044 |
| 148 | Precuneous Cortex (R) | 2 | -56 | 30 | 0.032 |
| 9 | Precuneous Cortex (L) | -8 | -48 | 60 | 0.045 |
| 135 | Vermis VI | 2 | -60 | -26 | 0.034 |
| 117 | Inferior Frontal Gyrus, pars triangularis (R) | 58 | 32 | 12 | 0.032 |
| 25 | Inferior Frontal Gyrus, pars opercularis (R) | 36 | 14 | 24 | 0.043 |
| 82 | Postcentral Gyrus (R) | 46 | -18 | 60 | 0.030 |
| 33 | Postcentral Gyrus (R) | 38 | -22 | 48 | 0.042 |
| 24 | Postcentral Gyrus (R) | 20 | -40 | 70 | 0.043 |
| 35 | Postcentral Gyrus (L) | -24 | -32 | 58 | 0.041 |
| 74 | Temporal Fusiform Cortex, posterior division (L) | -30 | -12 | -40 | 0.039 |
| 64 | Temporal Fusiform Cortex, posterior division (R) | 44 | -32 | -12 | 0.034 |
| 41 | Temporal Occipital Fusiform Cortex (L) | -42 | -56 | -16 | 0.036 |
| 57 | Inferior Temporal Gyrus, temporooccipital part (R) | 50 | -54 | -26 | 0.039 |
| 53 | Angular Gyrus (R) | 64 | -56 | 16 | 0.039 |
| 43 | Occipital Fusiform Gyrus (L) | -38 | -76 | -24 | 0.040 |
| 33 | WM Callosal body (R) | 16 | 32 | -6 | 0.042 |
| 31 | Lateral Occipital Cortex, superior division (L) | -46 | -72 | 22 | 0.042 |
| 28 | Juxtapositional Lobule Cortex (R) | 2 | -6 | 70 | 0.043 |
| 21 | Parahippocampal Gyrus, anterior division (R) | 14 | 2 | -40 | 0.043 |
| 20 | Thalamus (R) | 2 | -20 | 10 | 0.043 |
| 20 | Thalamus (L) | -8 | -28 | 6 | 0.043 |
| 20 | Brain-Stem | -12 | -36 | -24 | 0.041 |
| 18 | Putamen (R) | 26 | -14 | 14 | 0.044 |
| 17 | WM Callosal body (R) | 14 | 6 | 26 | 0.044 |
| 14 | Heschl's Gyrus (includes H1 and H2) (R) | 42 | -20 | 6 | 0.044 |
| 12 | Lingual Gyrus (R) | 12 | -88 | -12 | 0.044 |
| 11 | Lingual Gyrus (R) | 16 | -72 | -14 | 0.044 |

As a result of the highly significant superficial-cortical main effects in ASD and controls, Additional file 4 fails to represent the entire range of superficial-cortical activation patterns. To provide additional atlas information, we manipulated the significance thresholds for (SF Left Controls) left superficial amygdala correlations in controls using alpha 0.01 (p < 0.01, FWE corrected), (SF Left ASD) left superficial amygdala correlations in ASD using alpha 0.007 (p < 0.007, FWE corrected) and (SF Right ASD) right superficial amygdala correlations in ASD using a lower threshold of 0.01 and an upper threshold of 0.005 (0.01 > p < 0.05, FWE corrected).
