# Supplementary material for: Altered functional connectivity of the amygdaloid input nuclei in adolescents and young adults with autism spectrum disorder: a resting state fMRI study
Source: Mol Autism. 2016 Jan 28;7:13. doi: 10.1186/s13229-015-0060-x (PMC4730628; doi:10.1186/s13229-015-0060-x)
Supplement: Additional file 7: — Functional overlap between the superficial, laterobasal, and centromedial subregions in control subjects. (DOC 30 kb) [file 13229_2015_60_MOESM7_ESM.doc]

**Additional file 7. Functional overlap between the superficial, laterobasal and centromedial subregions in control subjects.**

|  | **Left** | | **Right** | |
| --- | --- | --- | --- | --- |
| **Nuclei** | *r* | *p* | *r* | *p* |
| SF - LB | .064 | .201 | .165** | .001 |
| CM - LB | -.109* | .030 | .179** | .000 |
| SF - CM | .114* | .023 | .122* | .016 |

All of the right amygdala subcompartments show significant positive correlations with each other. Left (SF - CM) superficial and centromedial activations show significant overlap, while left (CM - LB) laterobasal amygdala correlates negatively with the left centromedial amygdala. Left (SF – LB) superficial and laterobasal connectivity does however not overlap significantly. **Method:** Cortical target mean time series of subregion specific amygdala correlations were extracted using *fsl_sbca*. Colinearity between all subregions was assessed with two-tailed Pearson correlations analysis in SPSS (version 20) using alpha of 0.05. Number of volumes = 395; SF – LB = shows Pearson’s correlation coefficient (*r*) and its level of significance (*p*) between the superficial and laterobasal subcompartment; CM – LB = *r* and *p* between centromedial and laterobasal subcompartment; SF – CM = *r* and *p* between superficial and centromedial subcompartment.

**. Correlation is significant at the 0.01 level (2-tailed).

*. Correlation is significant at the 0.05 level (2-tailed).
