# Supplementary material for: Altered functional connectivity of the amygdaloid input nuclei in adolescents and young adults with autism spectrum disorder: a resting state fMRI study
Source: Mol Autism. 2016 Jan 28;7:13. doi: 10.1186/s13229-015-0060-x (PMC4730628; doi:10.1186/s13229-015-0060-x)
Supplement: Additional file 8: — Reduced functional connectivity in ASD. Demonstrates between-group differences of entire amygdalo-cortical correlations and subregion-specific partial correlations between participants with autism spectrum disorder and control subjects. (DOC 107 kb) [file 13229_2015_60_MOESM8_ESM.doc]

**Additional file 8. Reduced functional connectivity in ASD.**

| ***Reduced EA Connectivity in ASD*** | | | | | | |
| --- | --- | --- | --- | --- | --- | --- |
| Seed | Cluster Size | Structure | x | y | z | p-value |
| *Left EA* | 379 | Occipital Pole (L) | -24 | -90 | 34 | 0.026 |
|  |  |  | -18 | -92 | 38 | 0.031 |
|  |  |  | -16 | -90 | 42 | 0.034 |
|  |  | Cuneal Cortex (L) | -8 | -88 | 30 | 0.035 |
|  |  | Cuneal Cortex (R) | 6 | -70 | 20 | 0.047 |
|  |  |  | 12 | -72 | 22 | 0.038 |
|  |  | Supracalcarine Cortex (L) | -2 | -72 | 20 | 0.037 |
|  |  | Intracalcarine Cortex (L) | -2 | -70 | 14 | 0.038 |
|  | 35 | Lateral Occipital Cortex (L) | -26 | -60 | 58 | 0.040 |
|  |  | Superior Parietal Lobule (L) | -28 | -56 | 50 | 0.043 |
|  | 31 | Lateral Occipital Cortex (L) | -20 | -82 | 48 | 0.042 |
|  | 2 | Lateral Occipital Cortex (R) | 28 | -58 | 64 | 0.049 |
| *Right EA* | 56 | Superior Parietal Lobule (R) | 14 | -54 | 62 | 0.023 |
|  |  |  |  |  |  |  |
| ***Reduced Nucleus Connectivity in ASD*** | | | | | | |
| Seed | Cluster Size | Structure | x | y | z | p-value |
| *Left SF* | 2961 | Precuneous Cortex (R) | 16 | -58 | 8 | 0.003 |
|  |  | Cuneal Cortex (L) | -20 | -70 | 24 | 0.013 |
|  |  |  | -10 | -80 | 26 | 0.010 |
|  |  | Cuneal Cortex (R) | 18 | -78 | 34 | 0.011 |
|  |  |  | 6 | -70 | 22 | 0.004 |
|  |  | Supracalcarine Cortex (R) | 24 | -60 | 18 | 0.004 |
|  |  | Precuneous Cortex (R) | 12 | -62 | 18 | 0.004 |
|  |  |  | 18 | -56 | 18 | 0.005 |
|  |  | Precuneous Cortex (L) | -18 | -58 | 10 | 0.013 |
|  |  |  | -16 | -58 | 4 | 0.015 |
|  |  | Cuneal Cortex (L) | -2 | -72 | 22 | 0.005 |
|  |  | Occipital Pole (R) | 10 | -88 | 40 | 0.006 |
|  |  | Intracalcarine Cortex (R) | 6 | -62 | 12 | 0.006 |
|  |  | Intracalcarine Cortex (L) | -14 | -64 | 6 | 0.015 |
|  |  |  | -6 | -64 | 12 | 0.011 |
|  |  |  | -6 | -64 | 8 | 0.011 |
|  |  | Lateral Occipital Cortex (L) | -12 | -88 | 36 | 0.008 |
|  |  |  | -58 | -62 | -8 | 0.011 |
|  |  |  | -48 | -80 | 10 | 0.013 |
|  |  |  | -46 | -82 | 4 | 0.013 |
|  | 398 | Superior Parietal Lobule (R) | 28 | -54 | 62 | 0.004 |
|  |  |  | 32 | -54 | 70 | 0.005 |
|  |  |  | 40 | -44 | 68 | 0.035 |
|  |  |  | 38 | -40 | 64 | 0.039 |
|  |  | Postcentral Gyrus (R) | 38 | -30 | 68 | 0.023 |
|  | 368 | Lateral Occipital Cortex (R) | 44 | -60 | 12 | 0.023 |
|  |  |  | 42 | -70 | 14 | 0.023 |
|  |  |  | 44 | -68 | 28 | 0.033 |
|  |  |  | 40 | -72 | 26 | 0.033 |
|  |  |  | 44 | -72 | 26 | 0.033 |
|  |  |  | 30 | -76 | 18 | 0.038 |
|  |  | Angular Gyrus (R) | 52 | -58 | 18 | 0.032 |
|  |  |  | 44 | -56 | 24 | 0.036 |
|  | 10 | Precentral Gyrus (R) | 44 | -16 | 64 | 0.047 |
| *Right LB* | 12 | Superior Parietal Lobule (R) | 14 | -52 | 64 | 0.047 |

Reported cluster peaks and local maxima indicate areas of higher (Reduced EA connectivity in ASD) entire amygdalo-cortical correlations in controls compared to the ASD group between (Left EA) left entire amygdala and the listed structures and (Right EA) right entire amygdala correlations and the listed structures. Higher (Reduced nucleus connectivity in ASD) subregion specific partial correlations are listed between (Left SF) left superficial subregion and the listed structures and (Right LB) right laterobasal subregion with right superior parietal lobe in controls compared to the ASD group (p < 0.05, FWE corrected).
