# Supplementary material for: Altered functional connectivity of the amygdaloid input nuclei in adolescents and young adults with autism spectrum disorder: a resting state fMRI study
Source: Mol Autism. 2016 Jan 28;7:13. doi: 10.1186/s13229-015-0060-x (PMC4730628; doi:10.1186/s13229-015-0060-x)
Supplement: Additional file 9: — Functional connectivity with age effects. Demonstrates between-group differences of entire amygdalo-cortical correlations and nuclei group-specific partial correlations between participants with autism spectrum disorder and control subjects and negative main effects of age in the superficial subcompartment. (DOC 85 kb) [file 13229_2015_60_MOESM10_ESM.doc]

**Additional file 10. Subject-wise tSNR levels per nucleus group**

| **Participant** | **Right CM** | **Left CM** | **Right SF** | **Left SF** | **Right LB** | **Left LB** |
| --- | --- | --- | --- | --- | --- | --- |
| 1 | 35.86 | 42.84 | 45.58 | 27.47 | 36.62 | 30.40 |
| 2 | 39.98 | 27.91 | 34.15 | 31.61 | 34.76 | 35.71 |
| 3 | 45.40 | 36.21 | 49.03 | 45.86 | 43.45 | 43.51 |
| 4 | 31.95 | 48.31 | 41.39 | 38.11 | 41.73 | 35.60 |
| 5 | 34.04 | 32.05 | 34.83 | 31.27 | 32.03 | 34.09 |
| 6 | 29.09 | 32.93 | 31.84 | 36.41 | 40.93 | 34.82 |
| 7 | 34.70 | 34.95 | 36.24 | 40.11 | 38.59 | 40.52 |
| 8 | 44.61 | 35.64 | 36.25 | 34.83 | 37.12 | 36.81 |
| 9 | 41.56 | 35.52 | 33.79 | 32.12 | 36.72 | 34.83 |
| 10 | 27.93 | 28.70 | 31.46 | 26.28 | 27.00 | 25.81 |
| 11 | 33.41 | 33.48 | 33.78 | 36.21 | 34.01 | 34.74 |
| 12 | 42.17 | 29.81 | 37.94 | 33.14 | 33.41 | 31.21 |
| 13 | 41.50 | 42.13 | 45.42 | 43.69 | 43.32 | 35.85 |
| 14 | 50.37 | 50.74 | 54.52 | 42.54 | 45.30 | 38.88 |
| 15 | 40.34 | 36.67 | 32.16 | 36.26 | 42.63 | 42.16 |
| 16 | 52.14 | 55.22* | 43.27 | 45.21 | 48.32 | 47.03 |
| 17 | 42.76 | 28.57 | 40.76 | 34.49 | 39.93 | 42.65 |
| 18 | 45.53 | 41.61 | 49.60 | 40.92 | 40.09 | 36.38 |
| 19 | 32.03 | 41.22 | 40.61 | 35.70 | 41.33 | 32.75 |
| 20 | 45.55 | 45.08 | 45.04 | 32.72 | 37.23 | 38.08 |
| 21 | 32.39 | 44.10 | 37.07 | 42.75 | 22.43* | 29.97 |
| 22 | 50.88 | 41.92 | 48.98 | 37.46 | 46.62 | 43.01 |
| 23 | 60.63 | 54.83* | 49.79 | 45.73 | 48.97 | 40.93 |
| 24 | 48.49 | 33.77 | 43.91 | 42.86 | 42.53 | 43.91 |
| 25 | 32.57 | 33.95 | 36.57 | 33.45 | 34.03 | 33.68 |
| 26 | 42.18 | 31.43 | 46.75 | 33.28 | 40.43 | 38.06 |
| 27 | 35.32 | 45.22 | 42.86 | 32.64 | 39.42 | 38.37 |
| 28 | 46.06 | 41.76 | 42.57 | 45.00 | 44.04 | 39.98 |
| 29 | 32.51 | 39.68 | 32.34 | 36.51 | 40.05 | 38.83 |
| 30 | 50.19 | 48.67 | 44.11 | 38.46 | 44.70 | 43.70 |
| 31 | 40.83 | 41.20 | 44.95 | 41.77 | 41.65 | 42.31 |
| 32 | 34.03 | 38.36 | 35.26 | 34.44 | 35.78 | 45.46 |
| 33 | 39.52 | 34.91 | 39.88 | 32.41 | 39.65 | 39.95 |
| 34 | 36.34 | 41.78 | 38.66 | 41.24 | 34.25 | 31.97 |
| 35 | 29.71 | 35.47 | 35.22 | 39.31 | 32.72 | 34.95 |
| 36 | 31.94 | 43.29 | 45.17 | 32.18 | 33.74 | 32.42 |
| 37 | 43.10 | 35.55 | 40.70 | 35.82 | 39.42 | 36.69 |
| 38 | 28.87 | 40.38 | 32.60 | 35.87 | 42.45 | 37.99 |
| 39 | 37.34 | 39.35 | 41.41 | 38.19 | 40.59 | 37.76 |
| 40 | 25.17 | 32.18 | 27.05 | 27.91 | 29.11 | 29.57 |
| 41 | 46.40 | 47.96 | 37.52 | 35.88 | 42.41 | 37.39 |
| 42 | 47.87 | 40.62 | 33.90 | 34.86 | 36.10 | 36.13 |
| 43 | 38.80 | 43.39 | 38.69 | 34.87 | 36.62 | 35.88 |
| 44 | 45.76 | 40.04 | 43.46 | 38.64 | 50.43 | 42.07 |
| 45 | 41.80 | 39.03 | 50.65 | 45.84 | 45.31 | 48.19 |

Additional file 10 shows the subject-wise tSNR (time-series’ signal-to-noise ratios) levels for each nucleus group. ASD group = Participant 1-20; Control group = Participant 21-45.*outliers
