# Supplementary material for: Altered functional connectivity of the amygdaloid input nuclei in adolescents and young adults with autism spectrum disorder: a resting state fMRI study
Source: Mol Autism. 2016 Jan 28;7:13. doi: 10.1186/s13229-015-0060-x (PMC4730628; doi:10.1186/s13229-015-0060-x)
Supplement: Additional file 10: — Subject-wise tSNR levels per nucleus group. (DOC 4303 kb) [file 13229_2015_60_MOESM11_ESM.doc]

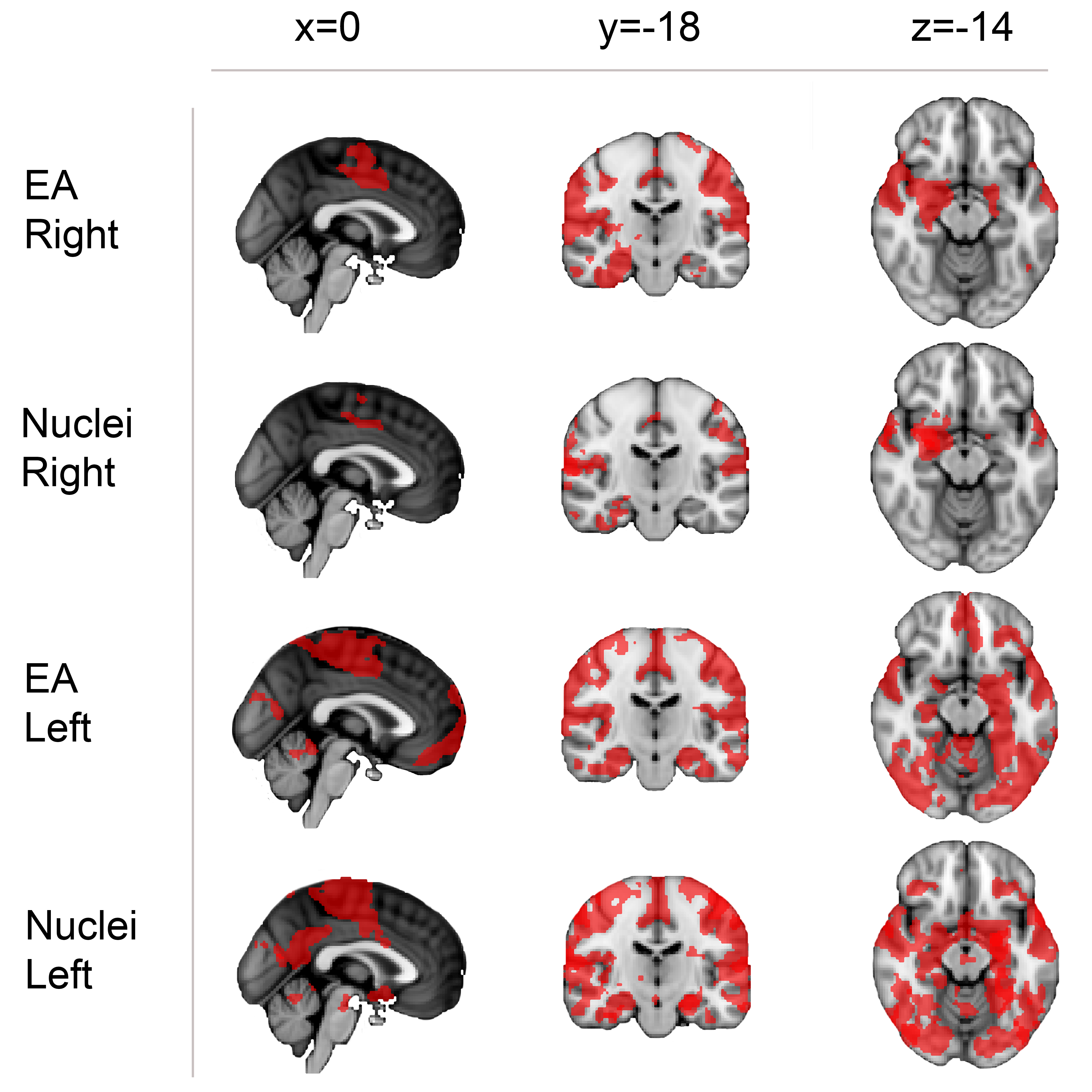


**Additional file 9. Similarity between all partial correlation maps combined and entire amygdala correlation maps in healthy subjects.**

This figure demonstrates the similarity between entire amygdalo-cortical connectivity and amygdalo-cortical subcompartment connectivity. The upper two rows depict intrinsic connectivity maps from (EA Right) the entire right amygdala seed compared to (Nuclei Right) all subcompartment partial correlation maps combined with overlay settings. The lower two rows show (EA Left) left entire amygdalo-cortical correlation maps and (Nuclei Left) all partial correlations subcompartment maps in the same way; (p < 0.05, FWE corrected).
