# Supplementary material for: Altered functional connectivity of the amygdaloid input nuclei in adolescents and young adults with autism spectrum disorder: a resting state fMRI study
Source: Mol Autism. 2016 Jan 28;7:13. doi: 10.1186/s13229-015-0060-x (PMC4730628; doi:10.1186/s13229-015-0060-x)
Supplement: Additional file 11: — Similarity between all partial correlation maps combined and entire amygdala correlation maps in healthy subjects. (DOC 55 kb) [file 13229_2015_60_MOESM9_ESM.doc]

**Additional file 9. Functional connectivity with age effects.**

| ***Reduced EA Connectivity in ASD*** | | | | | | |
| --- | --- | --- | --- | --- | --- | --- |
| Seed | Cluster Size | Structure | x | y | z | p-value |
| Left EA | 470 | Occipital Pole (L) | -24 | -90 | 34 | 0.025 |
|  | 66 | Lateral Occipital Cortex (L) | -26 | -60 | 58 | 0.036 |
|  | 42 | Lateral Occipital Cortex (L) | -20 | -82 | 48 | 0.041 |
|  | 13 | Lateral Occipital Cortex (L) | 28 | -58 | 64 | 0.045 |
|  | 9 | Lateral Occipital Cortex (L) | -24 | -70 | 54 | 0.047 |
| *Right EA* | 50 | Superior Parietal Lobule | 14 | -52 | 62 | 0.024 |
|  |  |  |  |  |  |  |
| ***Reduced Nuclei Connectivity in ASD*** | | | | | | |
| Seed | Cluster Size | Structure | x | y | z | p-value |
| *SF Left* | 3421 | Precuneous Cortex (R) | 18 | -58 | 8 | 0.002 |
|  | 531 | Superior Parietal Lobule (R) | 28 | -54 | 62 | 0.005 |
|  | 504 | Lateral Occipital Cortex (R) | 42 | -70 | 14 | 0.019 |
|  | 229 | Precuneous Cortex (R) | 2 | -62 | 64 | 0.025 |
|  | 127 | Lateral Occipital Cortex (L) | -26 | -70 | 62 | 0.036 |
|  | 41 | Superior Parietal Lobule (L) | -38 | -52 | 66 | 0.041 |
| *LB Right* | 14 | Superior Parietal Lobule | 14 | -52 | 64 | 0.054 |
|  |  |  |  |  |  |  |
|  | ***SF Left*** | | | | | |
|  | *negative age effect* | | | | | |
|  | Cluster Size | Structure | x | y | z | p-value |
|  | 33 | Temporal Pole (R) | 30 | 20 | -32 | 0.023 |
|  | 19 | Lateral Occipital Cortex, superior division (L) | -34 | -70 | 34 | 0.035 |
|  | 1 | Lateral Occipital Cortex, superior division (L) | -26 | -72 | 36 | 0.049 |

Additional file 9 shows functional connectivity between-group effects with age as covariate and negative main effects of age in left superficial nucleus. Cluster peaks indicate areas of higher (Reduced EA connectivity in ASD) entire amygdalo-cortical correlations in controls compared to the ASD group between (Left EA) left entire amygdala and the listed structures and (Right EA) right entire amygdala correlations and the listed structures with age as covariate. Higher (Reduced nucleus connectivity in ASD) nucleus specific partial correlations are listed between (Left SF) left superficial nucleus and the listed structures and (Right LB) right laterobasal nucleus with right superior parietal lobe in controls compared to the ASD group (left SF: p < 0.05, FWE corrected; right LB: p < 0.06, FWE corrected). Negative main effects of age are shown (SF Left) (p < 0.05, FWE corrected).
